# Time-trends in human urinary concentrations of phthalates and substitutes DEHT and DINCH in Asian and North American countries (2009–2019)

**DOI:** 10.1038/s41370-022-00441-w

**Published:** 2022-05-05

**Authors:** Elena Domínguez-Romero, Klára Komprdová, Jiří Kalina, Jos Bessems, Spyros Karakitsios, Dimosthenis A. Sarigiannis, Martin Scheringer

**Affiliations:** 1 RECETOX, Faculty of Science, Masaryk University, Kotlarska 2, Brno, 611 37 Czech Republic; 2 VITO (Flemish Institute for Technological Research), BE-2400 Mol, Belgium; 3 Aristotle Univ Thessaloniki, Dept Chem Engn, Environm Engn Lab, Univ Campus,Bldg D,Rm 201, Thessaloniki, 54124 Greece; 4 HERACLES Res Ctr Exposome & Hlth, Ctr Interdisciplinary Res & Innovat, Balkan Ctr, Bldg B,10thkm Thessaloniki Thermi Rd, Thessaloniki, 57001 Greece; 5 Sch Adv Study IUSS, Sci Technol & Soc Dept, Environm Hlth Engn, Piazza Vittoria 15, I-27100 Pavia, Italy

**Keywords:** Phthalate plasticizers, Phthalate substitutes, Time-trends, Human biomonitoring, Asia, North America.

## Abstract

**Background:**

Many phthalates are environmental pollutants and toxic to humans. Following phthalate regulations, human exposure to phthalates has globally decreased with time in European countries, the US and Korea. Conversely, exposure to their substitutes DEHT and/or DINCH has increased. In other countries, including China, little is known on the time-trends in human exposure to these plasticizers.

**Objective:**

We aimed to estimate time-trends in the urinary concentrations of phthalates, DEHT, and DINCH metabolites, in general population from non-European countries, in the last decade.

**Methods:**

We compiled human biomonitoring (HBM) data from 123 studies worldwide in a database termed “PhthaLit”. We analyzed time-trends in the urinary concentrations of the excreted metabolites of various phthalates as well as DEHT and DINCH per metabolite, age group, and country/region, in 2009–2019. Additionally, we compared urinary metabolites levels between continents.

**Results:**

We found solid time-trends in adults and/or children from the US, Canada, China and Taiwan. DEHP metabolites decreased in the US and Canada. Conversely in Asia, 5oxo- and 5OH-MEHP (DEHP metabolites) increased in Chinese children. For low-weight phthalates, the trends showed a mixed picture between metabolites and countries. Notably, MnBP (a DnBP metabolite) increased in China. The phthalate substitutes DEHT and DINCH markedly increased in the US.

**Significance:**

We addressed the major question of time-trends in human exposure to phthalates and their substitutes and compared the results in different countries worldwide.

**Impact:**

Phthalates account for more than 50% of the plasticizer world market. Because of their toxicity, some phthalates have been regulated. In turn, the consumption of non-phthalate substitutes, such as DEHT and DINCH, is growing. Currently, phthalates and their substitutes show high detection percentages in human urine. Concerning time-trends, several studies, mainly in Europe, show a global decrease in phthalate exposure, and an increase in the exposure to phthalate substitutes in the last decade. In this study, we address the important question of time-trends in human exposure to phthalates and their substitutes and compare the results in different countries worldwide.

## Graphical abstract

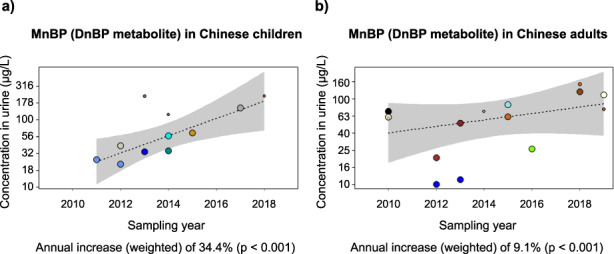

## Introduction

Phthalates are the most widely used plasticizers worldwide [1, 2]. Between countries, the consumption of plasticizers is led by China (approximately 50% of the world consumption in 2020), followed by other Asia/Pacific countries, Europe and North America [1]. Phthalates and major phthalate replacements such as 1,2-cyclohexanedicarboxylic acid, 1,2-diisononyl ester (DINCH) and di(2-ethylhexyl) terephthalate (DEHT) are principally used as plasticizers in polymers, principally in flexible PVC. Furthermore, low molecular weight phthalates (e.g. diethyl phthalate, DEP; di-isobutyl phthalate, DiBP; and di-n-butyl phthalate, DnBP) are used as solvents and/or plasticizers in adhesives, paints, lacquers, printing inks, and personal care products [2, 3]. In the plastics, phthalates are not chemically bound and can migrate to the environment, thus becoming environmental pollutants. From the environment, humans are exposed to these substances through different exposure routes (oral, dermal, inhalation) and sources [4–6]. Exposure to phthalates, depending on factors such as the exposure level and duration, may result in toxic outcomes in humans and animals. Notably, toxic effects of phthalates on the development and function of male and female reproductive systems [7–9], neurodevelopment [10–12], and metabolism [13] have been reported. Phthalate replacements such as DINCH and DEHT are also toxic [14–16]. Considering the toxicological properties of these substances, some phthalate uses have been regulated in different countries [17]. Importantly in the EU, fourteen phthalates have been classified as substances of very high concern (SVHC) in the Regulation (EC) No 1907/2006 on the Registration, Evaluation, Authorisation and Restriction of Chemicals (REACH), Annex XIV, by reason of their reproductive toxicity [18]. For each of these substances, a “sunset date” has been established, after which its placing on the market and use must be prohibited in general. Phthalates listed in Annex XIV include notably: di(2-ethylhexyl) phthalate (DEHP), butyl benzyl phthalate (BBP), DnBP, and DiBP, for which the sunset date was in February 2015. For these four substances (DEHP, DBP, BBP and DIBP), their endocrine disrupting properties were also included in REACH, Annex XIV, on November 23, 2021 [19, 20]. Moreover, dioctyl phthalate (DOP), diisononyl phtalate (DINP), and diisodecyl phthalate (DIDP) have been included in REACH, Annex XVII [21], and are subject to specific restrictions on the manufacture, placing on the market, and use.

Phthalate regulations help to reduce environmental as well as human exposure to these substances. Nonetheless, phthalates are presently still ubiquitous environmental pollutants. For example, phthalates have been found at high detection percentages (> 90%) in urine from the general population in Asian, European, and North American countries [22–24]. Considering the relatively short half-lives of this group of substances in humans, generally lower than 24-h in blood and urine [25], the high levels of detection in different countries are the result of recent exposure to these pollutants. Nevertheless, human exposure to phthalates seems to be globally decreasing. In this sense, the urinary concentrations of metabolites formed from low-weight phthalates and DEHP have decreased in the latest years in Germany [26, 27], Belgium [28, 29], Denmark [23], Sweden [30], Italy [31], the United States [32], Canada [33], and Korea [34]. Among high-weight phthalates, the urinary concentrations of DINP metabolites have diminished in Denmark [23] and Germany [27], and increased in Sweden [30]. Concerning phthalate replacements, metabolites from DEHT and/or DINCH increased in Denmark [23], Germany [35, 36], and the US [32]. Importantly, new time-trend analyses for European countries are conducted within the European Human Biomonitoring Initiative HBM4EU. More information on the time-trends of phthalates and phthalate replacements in the general population would be needed and would be helpful to interpret the effectiveness of various regulatory risk measurements in various parts of the world, like the authorisation and restriction in the EU as mentioned earlier, the concentration limits for children’s toys and childcare products and for food packaging in the US. Notably, there is a gap on the time-trends of these substances in the Chinese population [37].

Our objective was to analyse time-trends in the urinary concentrations of phthalates, DINCH, and DEHT in the general population from non-European countries (including China), in the last decade. We conducted a literature review and compiled human biomonitoring (HBM) data for phthalates and phthalate replacements for different age groups and countries worldwide. In summary, data from 123 publications for metabolites from all phthalates, DEHT and DINCH in more than 30 countries were compiled within the “PhthaLit” database. Based on the compiled central values (medians, geometric means), we analysed time-trends in urinary concentrations per metabolite, age group, and country/region, in non-European countries. Additionally, we compared phthalate urinary concentrations in adults and children, between continents (including Asia, North America, and Europe). We found significant time-trends for all the main phthalates in China, Taiwan, Korea, the US, and Canada, and for DEHT and DINCH metabolites in the US.

## Material and methods

### Literature research

We aimed to conduct a non-biased literature review to prepare a human biomonitoring (HBM) data compilation. In this sense, our literature research was in agreement with general recommendations for time-trend reviews [38]. We conducted the literature research on the Web of Science. The dates, research keywords, exclusion criteria, and results of the literature research are summarized in the first Supplementary Information file (SI 1), Table S1.

### “PhthaLit” database structure

We created the “PhthaLit” database as a detailed compilation of literature HBM data for phthalates, DEHT, and DINCH in the general population from different countries worldwide. The main columns in the database included variables considered of interest for the analysis of HBM data for phthalates. These variables are described below. General variables for traceability (reference, specific biomonitoring plan, and study identification number) are described in the section entitled: Data traceability in the “PhthaLit” database.

#### Substances and abbreviations

In the literature, a diversity of definitions and abbreviations exist for phthalates and their metabolites. The main abbreviations and definitions found in the literature for these substances and their metabolites are shown in SI 1, Table S2. We here used one abbreviation per substance. Among phthalate replacements, our priority was for DEHT and DINCH [39].

#### Phthalate concentrations in urine

In the current manuscript, we work on the central values of urinary concentrations of phthalates and phthalate replacements. As central values, for publications where medians and geometric means (GM) were reported, both values were compiled, due to the generally lognormal distribution of urinary phthalate concentrations [23, 27, 34]. However, only one of these metrics (priority for median as a more robust estimate of central tendency, followed by GM) per source was used for data analysis. In the current manuscript, the number of central values that we used in our analyses is identified by the abbreviation “*n*”. This abbreviation should not be confounded with “*N*”, which refers to the study-specific sample size (cf. Sample size (*N*)).

Concerning the units, unadjusted urinary concentrations (µg/L) were the most frequent data found in the literature, followed by data corrected for urinary dilution, in µg/g creatinine. Although we compiled data in the different units, we work on unadjusted urinary concentrations (µg/L) in the current manuscript. Besides, for one study which reported only creatinine-corrected data and showed the geometric mean of measured creatinine concentrations in the study population [40], we back-calculated the unadjusted concentrations (SI 1, section S3.1. Equation S1) and included them in the analysis. Additionally, for few publications which reported exclusively specific-gravity (SG) adjusted data, we considered the SG-adjusted data to be equivalent to unadjusted concentrations. In this sense, Runkel et al. (2020) [41] showed little differences between SG-adjusted and unadjusted phthalate concentrations in adults and children.

In the literature, some publications reported the central values of urinary concentrations for specific substances to be below the limits of detection (LOD, µg/L) or quantification (LOQ, µg/L). In those sources, censored data were generally corrected through substitution methods. Specifically, the values <LOD or <LOQ were generally replaced by LOD/2 or LOQ/2, respectively [23, 24]. Similarly, other authors replaced censored data by LOD/√2 or LOQ/√2 [32, 42]. Following the same substitution methods, we replaced the central values <LOD or <LOQ by LOD/2 and LOQ/2, respectively. More details are given in the SI 1, section S3.2. Substances with > 50% of censored central values in the database (see Fig. S1 for adults) were excluded from further data analyses.

#### Sampling years

We compiled data for sampling years from 2009 onwards.

In the literature, most studies reported data for one sampling campaign (in other words, for one time-point). Additionally, some “time-trend” studies (including cross-sectional studies with multiple time-points and longitudinal studies), which reported data for consecutive sampling campaigns (consecutive time-points), were also found. We compiled data from both types of studies. To be precise, in the reviewed HBM studies, one sampling campaign (i.e. one time-point) did not necessarily refer to one specific year but to one period (e.g. Aug. 2015–Sep. 2016; 2013–2015…). In our database, the average year per sampling time point was determined and used for our analyses. However, for studies with long sampling campaigns, we considered that an average sampling year may not be representative enough for the purposes of this study and could introduce an error in the time-trend analyses. In this sense, during data compilation, we decided to exclude data reported for sampling time points (specific periods) longer than 4 years.

#### Population and age groups

We aimed to compile HBM data which may be representative of the exposure levels for the general population in a country. Therefore, we did not collect data from more highly exposed groups, such as a) occupationally exposed workers, b) population from reported “hotspot” locations (i.e. including phthalate manipulation sites), and c) transfused patients. We also excluded data from subjects with confirmed or suspected reproductive pathology (e.g. couples undergoing infertility treatment) or chronic disease (e.g. obesity). For studies where a specific group of population, not representative for the general population, was compared with a control group, data from the controls were compiled. Finally, we did not compile data for pregnant women, since pregnancy may have an impact on phthalate pharmacokinetics [43].

We classified the data by age group [27, 44]. Specifically, the following groups were defined, depending on the average age (age) of the subjects: “adults” (18 years < age < 60 years), “children” (4 years <= age <= 12 years), “teenagers” (12 years < age <= 18 years), “seniors” (60 years <= age), and “younger children” (age < 4 years). The main age groups for which HBM data for phthalates were found were those of adults, followed by children (cf. Results, PhthaLit database). Therefore, we considered both age groups as a priority for our study.

#### Countries and continents

In the database, the continent, country, and region (if it was shown), were specified for each datum.

In Asia, several studies published HBM data specifically for the Taiwanese population [40, 45–47]. As a consequence the number of data available in Taiwan was relatively high (SI 1, Tables S3 and S4). For these reasons, we analysed Taiwan separately.

In Europe, time-trend analyses for these plasticizers are conducted within the HBM4EU project. Thus, we excluded European countries from our time-trend analyses (cf. Statistical methods, Time-trend analysis). Nevertheless, data from European countries were included in the “PhthaLit” database, and Europe was included for geographic comparisons of phthalate levels between continents (cf. Statistical methods, additional analyses).

#### Sample size (N)

In the database, per study, we specified the number of subjects who provided urine for phthalate measurements (i.e., study-specific sample size, “*N*”). This variable (*N*) was used for data weighting in our time-trend analysis (cf. Statistical methods, Time-trend analysis). No data were compiled for *N* < 20.

#### Other variables

Major variables explained above (i.e. measured substance, age group, country, and sampling year) created a high data categorization. For this reason, other variables which were considered comparatively less important for our analysis, for example the gender, area (urban,  suburban), or the sample type were not included as variables for time-trend calculations per country or geographic comparisons per continent. Nevertheless, these variables were specified in the database and we analysed the possible impact of the gender, sample type, and area on the estimated trends (cf. Statistical methods, additional analyses).

#### Data traceability in the “PhthaLit” database

In the database, general variables for traceability were included, particularly:

- Reference short name and digital object identifier (DOI).
- Human biomonitoring (HBM) survey name.
- Study identification number (Study ID number). This number identifies data from the same source. Generally, one Study ID number identifies data from one individual study. Nevertheless, for some studies which reported data from the same HBM survey in a population, we used the same Study ID number (see SI 1, Tables S5 and S6). This number was used for data analysis (e.g. to calculate the median value if the same study reported separate data in male and female) and for the identification, in the Figures, of data from the same source (same colour).

### Statistical methods

#### Time-trend analysis

In order to calculate time-trends between 2009 and 2019, the central values in the database were aggregated per country, age group, metabolite, year, and study (Study ID number, see Data traceability in the “PhthaLit” database), providing one median value for each unique combination of these variables. This principle was applied to all data, including for studies which reported only separated data for variables not included in the analysis (e.g. separated data for men and women, or for different cities/areas), for which the median value per country, age group, metabolite, year, and study/survey was calculated from all data and used in the time-trend analysis. Each median was also characterised by the sum of sample sizes used for its computation. The study-specific sample size (*N*) was used to assign an importance to each median value within the analysis (weighting). A minimum sample size of 120 has been proposed for derivation of European Reference Values in a population, in order to enable a proper estimation of the 95% CI of the 95th percentile [48–51]. Taking this value as a reference, we considered that central values (medians or geometric means) computed from 120 or more subjects from the general population per study group were fully representative. Therefore, depending on *N*, we assigned a weight to each central value:

- for studies with *N* >=120, the weight of the central values was 1;
- for studies with *N* < 120 (i.e., 20 <= *N* < 120), the weight of the central values was ⌈N/120⌉, where the half-brackets ⌈⌉ represent the ceiling operation (rounding up).

Assuming an approximately log-normal distribution of the data, we logarithmically (ln) transformed the urinary concentrations, similarly to the approach by Frederiksen et al. [23]. Next, we looked for linear time-trends on these log-transformed data. Time-trends were investigated using the non-parametric Kendall test and the Theil-Sen trend estimator [48]. These robust methods are not prone to be biased by possible extreme values and provide robust estimates of the presence and slope of the trend. Specifically, we applied these methods for a) time series including 5 or more central values (“*n*” ≥ 5), and for b) time-series with lower *n* (*n* = 3–4) only if data from the national human biomonitoring plan in the country were available at the earliest and the latest years in the time-series. Results with *p* ≤ 0.10 were considered significant.

#### Additional analyses

We analysed the possible impact of the gender, area, and sample type on the estimated trends per metabolite, country, and age group. Specifically, all time-trend analyses were conducted separately per gender, area, and sample type, and compared to the original trends using Fisher z-scoring [52]. None of these variables had a significant impact in any trend. The *p*-value of the trend difference test [52] was in all cases higher than 0.10. Further details are shown in the SI 1 (section S3.3).

Furthermore, geographic comparisons in the urinary phthalate concentrations between continents were conducted. Specifically, the central values (i.e., median, GM) of urinary concentrations per metabolite, continent, and age group, for two different periods (i.e., 2009–2014 and 2015–2019) were compared. Further details are given in the SI 1 (section S3.4).

## Results

### “PhthaLit” database

The “PhthaLit” database, generated and analysed during this study, can be found as an Excel file of Supplementary Information (SI 2) with the current manuscript.

Data from 123 publications were compiled, including principally peer-reviewed publications, as well as data reported by the US Centers for Disease Control and Prevention [42], Health Canada [24], and Santé Publique France [53]. In summary, 2287 compiled central values of unadjusted urinary concentrations (µg/L) of phthalates and phthalate replacements from the “PhthaLit” database were available and used in the time-trend analysis. Concerning the distribution of data among age groups, more unadjusted central values were found for adults (*n* = 1208) and children (*n* = 859), than for teenagers (*n* = 160) or other age groups. Between countries, a high number of data were found principally in China, followed by Germany (also Denmark for adults) and the United States, both in adults and children. Among substances, more data were available for DEHP metabolites and low-weight phthalates than for high-weight phthalates and phthalate replacements (Tables S3 and S4).

### Time-trends

Significant time-trends per substance, country/region, and age group, in percentages of change per year, are summarized in Tables 1 and 2. In these Tables, the specific periods studied and data sources are reported. Only trends including <33% of censored data, one or more data points after 2015, for a total period >= 4 years are shown.

**Table 1:**
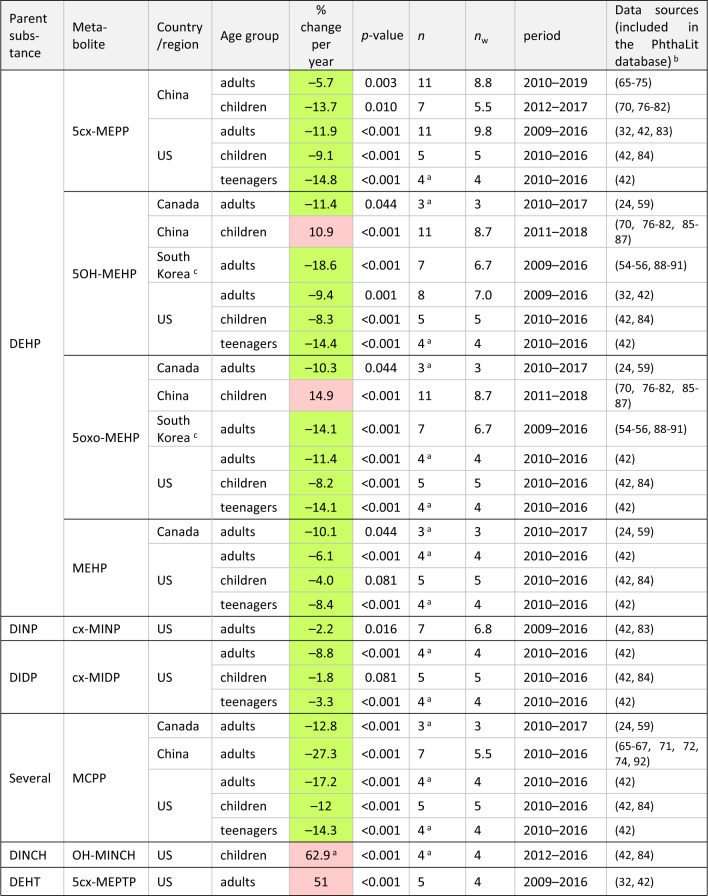
Significant time-trends for metabolites formed from DEHP, high-weight phthalates, DEHT and DINCH.

Green: decreasing trends; red: increasing trends. Only significant trends (*p*-value <= 0.10) including <33% of censored data, with one or more data points after 2015, for a total period >= 4 years are shown.

*n* = number of central values aggregated per country, age group, metabolite, year, and Study ID number, per time-trend; *n*_w_ = number of “full-weight” data per trend, calculated as the sum of the number of aggregated central values (*n*) multiplied by their weight (Statistical methods, time-trend analysis).

^a^For trends with *n* < 5 (i.e., trends in Canada and some trends in the US), data from national human biomonitoring plans [24, 42] were used both at the earliest and the latest years per trend.

^b^For studies where a specific group of population was compared with a control group, data from controls were compiled.

^c^Trends in Korea were overestimated (see Discussion).

**Table 2:**
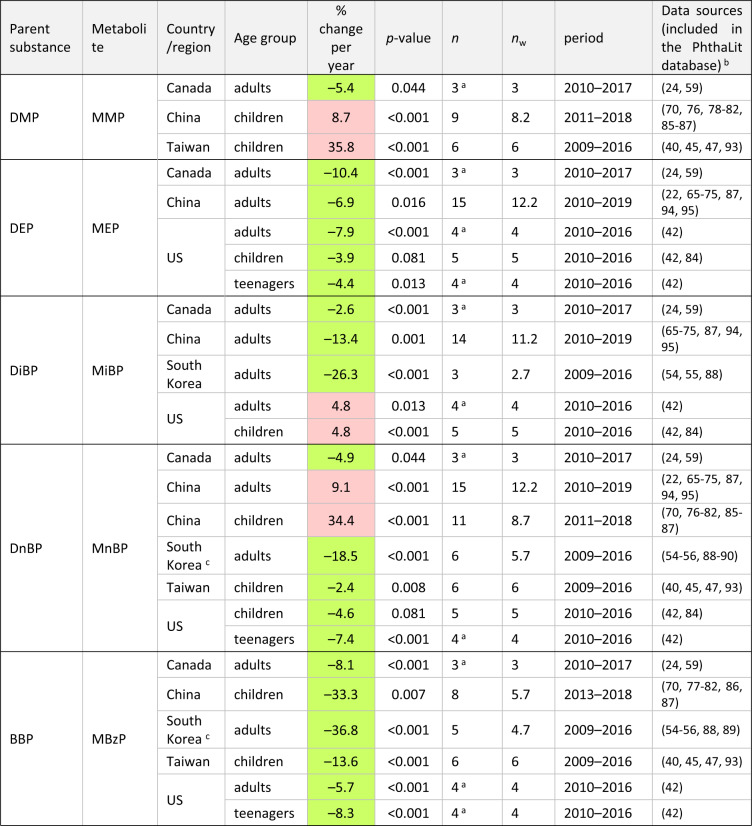
Significant time-trends for metabolites formed from low-weight phthalates.

Green: decreasing trends; red: increasing trends. Only significant trends (*p*-value < = 0.10) including <33% of censored data, with one or more data points after 2015, for a total period >= 4 years are shown.

*n* = number of central values aggregated per country, age group, metabolite, year, and Study ID number per time-trend; *n*_w_ = number of “full-weight” data per trend, calculated as the sum of the number of aggregated central values (*n*) multiplied by their weight (Statistical methods, time-trend analysis).

^a^For trends with *n* < 5 (i.e., trends in Canada and some trends in the US), data from national human biomonitoring plans [24, 42] were used both at the earliest and the latest years per trend.

^b^For studies where a specific group of population was compared with a control group, data from controls were compiled.

^c^Trends in Korea were overestimated (see Discussion).

Time-trends were found principally in adults and/or children from five countries/regions, including three in Asia (China, Taiwan, Korea) and two in North America (Canada and the US). Only 4 trends in US children, out of 58 significant trends, had *p*-values between 0.05 and 0.10. The other 54 trends were significant at *p* < 0.05 (see Tables 1 and 2). Time-trends included generally 5 or more central values. Exceptionally, trends in Canada and some trends in the US with a lower (*n* = 3–4) were also included, since data from the national human biomonitoring plans in those countries [24, 42] were available at the earliest and the latest sampling years per trend. Importantly, the trends in both North American countries were globally consistent.

#### DEHP metabolites

Significant time-trends were found for the main DEHP metabolites (Table 1).

**Fig. 1:**
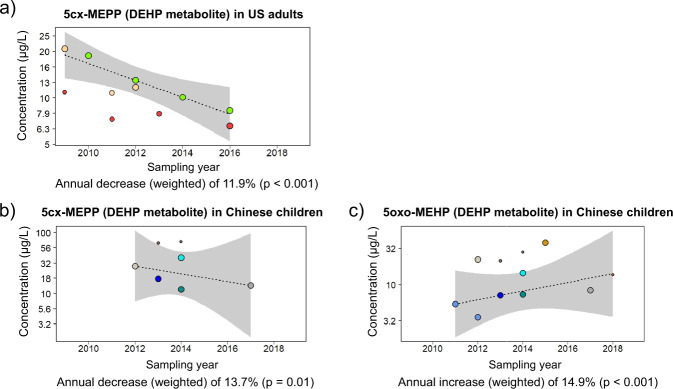
Time-trends in the urinary concentrations of DEHP metabolites (µg/L). **a** 5cx-MEPP in US adults. **b** 5cx-MEPP in Chinese children. **c** 5oxo-MEHP in Chinese children. Each symbol is a central value (i.e. median or geometric mean). The size of the symbols is related to the study-specific sample size (*N*) and its weight in the time-trend calculation (see Statistical methods, time-trend analysis). Data from the same data source (Study ID number: see Data traceability in the “PhthaLit” database) are identified by symbols with the same colour in all panels and figures. Grey area: 90% confidence interval.

In the US, Canada, and Korea, DEHP metabolites decreased with time. Notably in the US population and Canadian adults, the urinary concentrations of secondary DEHP metabolites consistently decreased by approximately 8–15% per year (Fig. 1a).

In China, time-trends for DEHP metabolites diverged between age groups and metabolites. On the one hand, decreasing trends were found for 5cx-MEPP in children (–13.7% yr^–1^; Fig. 1b) and adults (–5.7% yr^–1^) (Fig. S2a). On the other hand, 5oxo- and 5OH-MEHP increased by 11–15% per year in children (Figs. 1c and S3a). Data for 5oxo-MEHP and 5OH-MEHP in Chinese adults did not seem to follow a linear time-trend pattern, and no significant trends were found (Fig. S2b). The results for DEHP in China are further discussed below.

#### DINCH and DEHT metabolites

**Fig. 2:**
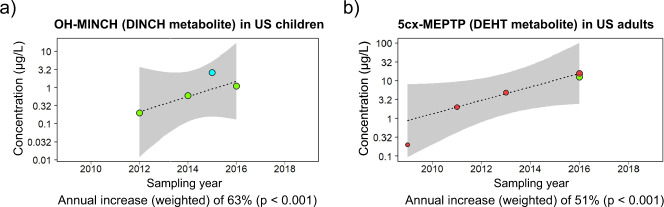
Time-trends in the urinary concentrations of phthalate replacement metabolites (µg/L). **a** OH-MINCH (a DINCH metabolite) in US children. **b** 5cx-MEPTP (a DEHT metabolite) in US adults. Each symbol is a central value (i.e. median or geometric mean). The size of the symbols is related to the study-specific sample size (*N*) and its weight in the time-trend calculation (see Statistical methods, time-trend analysis). Data from the same data source (Study ID number: see Data traceability in the “PhthaLit” database) are identified by symbols with the same colour in all panels and figures. Grey area: 90% confidence interval.

In the US population, phthalate replacements markedly increased with time. Specifically, significant increases were found for OH-MINCH in children (+62.9% yr^–1^) and 5cx-MEPTP in adults (+51.0% yr^–1^) (Fig. 2).

#### Low-weight phthalates (DMP, DEP, DnBP, DiBP, BBP)

For low-weight phthalates, contrasting results between countries/regions and substances were found (Table 2). In China, Taiwan, and the US, metabolites from some low-weight phthalates decreased while others increased. In Canada and Korea, low-weight phthalates decreased with time.

**Fig. 3:**
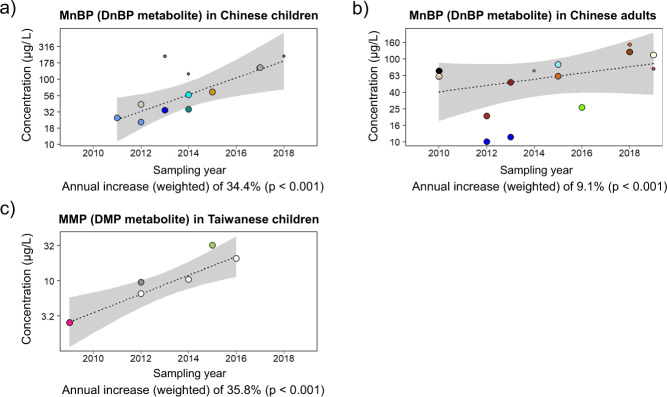
Time-trends in the urinary concentrations of low-weight phthalate metabolites (µg/L). **a** MnBP (DnBP metabolite) in Chinese children. **b** MnBP in Chinese adults. **c** MMP (DMP metabolite) in Taiwanese children. Each symbol is a central value (i.e. median or geometric mean). The size of the symbols is related to the study-specific sample size (*N*) and its weight in the time-trend calculation (see Statistical methods, time-trend analysis). Data from the same data source (Study ID number: see Data traceability in the “PhthaLit” database) are identified by symbols with the same colour in all panels and figures. Grey area: 90% confidence interval.

To begin with Asian countries/regions, MMP increased in children from Taiwan (+35.8% yr^–1^) and China (+8.7% yr^–1^) (Figs. 3c and S4a). Importantly, in China, MnBP consistently increased both in children (+34.4% yr^–1^) and adults (+9.1% yr^–1^) (Fig. 3a, b). Other low-weight phthalates decreased. Notably, MBzP consistently decreased in children from China (–33.3% yr^–1^) and Taiwan (–13.6% yr^–1^) (Fig. S4b, c), MiBP and MEP decreased in Chinese adults (–13.4% yr^–1^ and –6.9% yr^–1^, respectively) (Fig. S5).

In the US population and in Canadian adults, low-weight phthalate biomarkers generally decreased by approximately 3–10% yr^–1^ (Fig. S6a, c). In contrast, MiBP increased in US adults and children (+4.8% yr^–1^) (Fig. S6b).

#### High-weight phthalates (DINP, DIDP), and MCPP (non-specific metabolite)

For DINP and DIDP metabolites, specifically for cx-MINP and cx-MIDP, respectively, decreasing trends by 2–9% yr^–1^ were found in the US population (Table 1).

Concerning MCPP, this is a non-specific metabolite that can be formed through metabolism of several high- and low-weight phthalates [26]. We found decreasing trends for MCPP in the US, Canada, and China (Fig. S7).

## Discussion

We analysed time-trends in the urinary concentrations of phthalates in general population from Asian and North American countries, following a literature review and data compilation. Naturally, analysing data from multiple sources presented a challenge based on the significant inter-study variability underlying the data sets. To deal with this matter, it was essential to a) conduct a non-biased literature review and a thorough data compilation, and b) select the main variables for the time-trend analyses. As major variables, we included the metabolite, age group, country, sampling year, and sample size. By including the major variables in our analysis, on the one hand we found globally solid time-trends. On the other hand, this created a high data categorization. As an illustration, significant trends per metabolite, age group, and country were found “just” in 5 non-European countries/regions, and a similar number of European countries (not shown), out of approximately 30 countries for which data were compiled. To avoid further data categorization, other variables (i.e., gender, sample type, area) were not included in the analyses. Importantly, we tested the possible impact of those excluded variables (gender, area, sample type) on the estimated time-trends. None of these variables had a significant impact in any trend (SI 1, section S3.3). Nevertheless, we found a bias for the time-trends in Korea, for different reasons. Specifically in Korea, phthalate concentrations reported in 2016 by Lee I. et al. and Lee G. et al. [54, 55] were low, as compared to KoNEHS data [56] (see Fig. S3b). Importantly, Lee I. and Lee G. et al. measured phthalates in spot urine collected in control women, following a fasting of at least 8 h [54, 55]. Fasting prior to the collection of spot urine (which was not first morning urine) was relatively rare in the reviewed literature for phthalates. For instance, no other studies with this design were included in the “PhthaLit” database. Importantly, fasting may reduce the exposure to these substances. In fact, the diet is considered as a major exposure source for certain phthalates [4–6]. Thus, the use of spot urine data following fasting may have biased the trends in Korea, which were overestimated, as compared to less pronounced decreasing trends suggested by KoNEHS data [34, 56]. Importantly, no bias was found for the time-trends in China, Taiwan, the US, and Canada.

A question remains concerning some inconsistent time-trends between DEHP metabolites in China. To clarify this question, we need to mention a particularity of the data in China. Specifically in this country, we found more HBM data for DEHP and low-weight phthalates, from a higher number of peer-reviewed studies, than in any other country. Conversely, we found no data from cross-sectional national human biomonitoring studies in China. This is in opposition to other countries, for which phthalate data from national HBM studies were found (e.g. the US National Health and Nutrition Examination Survey, NHANES; the Canadian Health Measures Survey, CHMS; and the Korean National Environmental Health Survey, KoNEHS) and included in the time-trends estimations. Importantly, the China National Human Biomonitoring (CNHBM) survey started in 2017–2018 [57], and data from this survey may facilitate future time-trend analyses in this country. In our study, data in China presented quite large inter-study variability. Hence, the number of data available per time-trend was particularly important in this country. Specifically, to identify the most reliable time-trends in China, we analysed the number of central values (*n*) for the whole study period and for the most recent years (i.e. since 2015), per trend. In this sense, among DEHP metabolites, we found more data for 5oxo-MEHP and 5OH-MEHP than for 5cx-MEPP, both in adults and children (cf. Figs. 1b, c and S2). In adults, log-transformed data for 5oxo- and 5OH-MEHP did not follow a linear time-trend pattern and no significant trends were found. Conversely in children, we found significant time-trends for 5OH- and 5oxo-MEHP (*p* < 0.001), which were more solid than those for 5cx-MEPP (*p* = 0.010). To summarize our results for DEHP metabolites in China, we found that 5OH-MEHP and 5oxo-MEHP increased over time, while 5cx-MEPP seemed to decrease over time, which is unexpected since the three of them are metabolites of DEHP. Notably, this apparent decreasing time trend for 5cx-MEPP could be due to chemical-analytical flaws. It has been observed that 5cx-MEPP may co-elute with OH-MINP (oral communication Dr Koch). OH-MINP being a metabolite of the high molecular weight phthalate DINP might be decreasing and so cause confusion where the quantified peaks may be attributed (partly) mistakenly to 5cx-MEPP instead of OH-MINP. Concerning the trends for low-weight phthalates in China, a relatively high number of central values per metabolite and age group (*n* = 11–15) were available for the time-trend calculations for MnBP (adults and children), MEP and MiBP in adults, which supports the solidity of these results (i.e. increasing trends for MnBP and decreasing trends for MEP and MiBP). Importantly, MnBP consistently increased in adults and children. This consistency between age groups highlights the reliability of these results, since the majority of data sources were independent between adults and children (i.e., for MnBP, out of 11–15 data sources per age group, only 2 sources were common to both age groups). For MMP and MBzP in Chinese children, a lower number of central values was found (*n* = 8–9). Interestingly for these two metabolites, we found consistently increasing trends for MMP and decreasing trends for MBzP both in Chinese and Taiwanese children.

To sum up, in the general populations from the US and Canada, phthalate metabolites generally decreased with time. Conversely, metabolites from phthalate replacements (DEHT, DINCH) increased in the US. Globally, these results were in agreement with decreasing trends for phthalate metabolites, and increasing trends for metabolites of the substitutes DEHT and DINCH in population from Puerto Rico [58], Germany [26, 27, 35, 36], and Denmark [23]. In Canadian population, phthalate time-trends have been recently reported by Pollock et al., 2021 [33]. Although there is a partial overlap in data sources used by Pollock et al. (2021) and those used in the current study, we additionally used data by Albert et al. 2018 [59]. Other differences between our data analysis in Canada and the study by Pollock et al. (2021) are for the time periods considered, the age ranges, and the selection of metabolites. The upshot is that Pollock et al. [33] showed decreasing trends for DEHP metabolites, low-weight phthalate metabolites (specifically, MEP, MnBP, and MBzP), and MCPP in Canadian population, which is consistent with our results. In addition, we found slight decreasing trends for MMP and MiBP in this country (these metabolites were not assessed in the study by Pollock et al. [33]). Interestingly, for low-weight phthalates, we found diverging time-trends between substances in China, Taiwan, and the US. In other words, in these countries/regions some low-weight phthalates increased while others decreased. These results might be related to a substitution in the uses of certain low-weight phthalates by others. Notably, we found contrasting trends between DnBP and DiBP monoesters in the US and China. For example, in the US, MiBP increased while MnBP decreased with time. Similarly, in men attending a fertility clinic in Boston between 2000 and 2017, MiBP increased while other phthalates generally decreased [60]. In China, we globally found solid trends, excepted for 5cx-MEPP for which chemical analytical flaws could not be excluded. The results in Chinese population suggested notably an increasing exposure to DEHP, DnBP and DMP, and decreasing exposure to DEP and DiBP in China in the last decade. The increases in the urinary concentrations of DEHP metabolites in Chinese population contrasted with decreasing trends in North American and European countries. Interestingly, in Japanese children, the concentrations of phthalate metabolites (including DEHP metabolites) were stable between 2012 and 2017 [61]. To finalize, it should be noted that the increases for MnBP in Chinese population, MMP and 5OH-MEHP in Asian children occurred in a context where the average concentrations of these substances in the last years seemed to be relatively high in Asia, as compared to data in other continents (SI 1, section S3.4). In conclusion, our results in China raise some concern and seem globally consistent with the leading position of Asia in the plasticizer global market, and the increasing importance of China in this market (i.e. China represented approximately 42% of the worldwide consumption of plasticizers in 2017, and 50% in 2020) [1, 62].

To conclude with few recommendations for future research, more HBM data for DEHT and DINCH would be needed worldwide. The quite large increase per year of DEHT and DINCH metabolites in the US is quite troublesome because of the recent knowledge on the toxicological properties that also these phthalate substitutes exhibit. Furthermore, the world consumption of phthalate replacements is predicted to increase markedly in the following years (2021–2025) [1]. For high-weight phthalates, more data would be desirable too, with a focus on the secondary metabolites from these substances [63]. To finalize, environmental pollutants such as the studied plasticizers are a global concern. In this context, as previously suggested [64], the scarcity of HBM data for phthalates, DEHT, and DINCH in general population in some continents (Africa, South America, Oceania) and some countries worldwide was striking.

## Supplementary Information

Supplementary Information

Supplementary database

## Data Availability

The “PhthaLit” database, generated and analysed during this study, can be found as an Excel file of Supplementary Information (SI 2) with the current manuscript.
