# Supplementary material for: Time-trends in human urinary concentrations of phthalates and substitutes DEHT and DINCH in Asian and North American countries (2009–2019)
Source: J Expo Sci Environ Epidemiol. 2022 May 5;33(2):244–54. doi: 10.1038/s41370-022-00441-w (PMC10005949; doi:10.1038/s41370-022-00441-w)
Supplement: Supplementary file 1 — Supplementary Information [file 41370_2022_441_MOESM1_ESM.pdf]

Supplementary Information (SI 1) for:

Time-trends in human urinary concentrations of phthalates and substitutes DEHT and DINCH in Asian and North American countries (2009–2019)

Elena Domínguez-Romero[1]*, Klára Komprdová[1], Jiří Kalina[1], Jos Bessems[2], Spyros Karakitsios[3,4], Dimosthenis A. Sarigiannis[3,4,5], Martin Scheringer[1]

[1]RECETOX, Faculty of Science, Masaryk University, Kotlarska 2, 611 37 Brno, Czech Republic

[2]VITO Flemish Institute for Technological Research, BE-2400, Mol, Belgium

[3]Aristotle Univ Thessaloniki, Dept Chem Engn, Environm Engn Lab, Univ Campus,Bldg D,Rm 201, Thessaloniki 54124, Greece

[4]HERACLES Res Ctr Exposome & Hlth, Ctr Interdisciplinary Res & Innovat, Balkan Ctr, Bldg B,10thkm Thessaloniki Thermi Rd, Thessaloniki 57001, Greece

[5] Sch Adv Study IUSS, Sci Technol & Soc Dept, Environm Hlth Engn, Piazza Vittoria 15, I-27100 Pavia, Italy

**Corresponding author: Elena Domínguez-Romero. E-mail address:* elena.dominguez-romero@recetox.muni.cz

Table of Contents

S1. Supplementary Tables **4**
S2. Supplementary Figures **17**
S3. Additional information **26**
S3.1. Urinary concentration units 26
S3.2. Correction of censored data 26
S3.3. Impact of the gender, area, and sample type on the time-trends 26
S3.4. Geographic comparisons 27
References **28**

List of Supplementary Tables

Table S 1: literature search on the Web of Science, research keywords and exclusion criteria, and summary of results 4
Table S 2: list of abbreviation/s and definition/s of the parent substances and their metabolite/s found in the literature for phthalates and replacing plasticizers 6
Table S 3: number of central values (priority for median, followed by geometric mean) of unadjusted phthalate urinary concentrations per metabolite and country/region in adults, included in the "PhthaLit" database 9
Table S 4: number of central values (priority for median, followed by geometric mean) of unadjusted phthalate urinary concentrations per metabolite and country/region in children, included in the "PhthaLit" database 11
Table S 5: studies that shared a given Study ID number in the database (data for non-European countries) 12
Table S 6: studies that shared a given Study ID number in the database (data for European countries) 14

List of Supplementary Figures

Figure S 1. Number of measured (>LOD/LOQ, green) and censored (<LOD/LOQ, orange) central values per measured substance in adults, compiled in the "PhthaLit" database 17
Figure S 2: time-trend in the urinary concentrations of 5cx-MEPP (DEHP metabolite, µg/L) in Chinese adults (panel a); available data for 5oxo-MEHP (DEHP metabolite) in Chinese adults (panel b) 17
Figure S 3: time-trends in the urinary concentrations (µg/L) of the DEHP metabolite 5OH-MEHP in Chinese children (panel a) and Korean adults (panel b) 18
Figure S 4: time-trends in the urinary concentrations (µg/L) of: MMP (DMP metabolite) in Chinese children (panel a); MBzP (BBP metabolite) in Chinese children (panel b) and in Taiwanese children (panel c) 18
Figure S 5: time-trends in the urinary concentrations (µg/L) of MiBP (DiBP metabolite) and MEP (DEP metabolite) in Chinese adults (panels a and b, respectively) 19

Figure S 6: time-trends in the urinary concentrations (µg/L) of: MnBP (DnBP metabolite) and MiBP (DiBP metabolite) in US children (panels a and b, respectively); MBzP (BBP metabolite) in Canadian adults (panel c) 19
Figure S 7: time-trends in the urinary concentrations (µg/L) of MCPP in adults from Canada (panel a) and China (panel b) 20
Figure S 8: time-trends for MnBP in Chinese children: a) including all data; b) per sample type (spot urine) 20
Figure S 9: significant time-trends for MnBP in Chinese children per gender (a, both genders; b, girls; c, boys) 21
Figure S 10: significant time-trends for MnBP in Chinese children per area (a, mixed areas; b, suburban; c, urban) 21
Figure S 11: time-trends for 5OH-MEHP in Chinese children: a) including all data; b) for data measured in spot urine; c) for data measured in morning urine 22
Figure S 12: significant time-trends for 5OH-MEHP in Chinese children per gender (a, both genders; b, girls; c, boys) 23
Figure S 13: significant time-trends for 5OH-MEHP in Chinese children per area (a, mixed areas; b, suburban; c, urban) 23
Figure S 14: boxplots of urinary concentrations (µg/L) of low-weight phthalates per continent and period (2009–2014 vs 2015–2019) in adults and children, for: MMP (a), MEP (b), MiBP (c), MBzP (d), and MnBP (e) 25
Figure S 15: boxplots of urinary concentrations (µg/L) per continent and period (2009–2014 vs 2015–2019) in adults and children, for: 5OH-MEHP (a), and MCPP (b). 25

S1. Supplementary Tables

Table S 1: literature search on the Web of Science, research keywords and exclusion criteria, and summary of results

**Research keywords**	**Date**	**Screening of results[a]**		**Number of publications pre-selected for data compilation**			**Number of publications used in data compilation and analysis**
		**Number of results**	**Direct exclusion criteria[a]**	**Total**	**After elimination of duplicates**	**Further exclusion criteria for pre-selected publications[b]**	
TOPIC: phthalate or phthalates or plasticizer or plasticizers<br>& TITLE: trends or trend	31/10/19	50	• Median sampling year earlier than 2009<br><br>• Data not representative of the general population (e.g. occupational exposure)<br><br>• Specific physiological stages: pregnancy, breastfeeding	7	131	Sampling year:<br>• Unclear sampling year (i.e. not shown, N=6)<br>• Very long sampling campaign (>4 y; N=2)<br><br>Data which may not be representative of general population in a country:<br>• Hotspot region (N=4)<br>• Subjects undergoing infertility study/treatment (N=14)<br>• Subjects with chronic health problems (N=3)<br>• Publications with low sample sizes were not prioritized. These publications were directly excluded if N<20 (N=6)<br><br>Other reasons:<br>• The HBM data shown were already compiled from other publications (N=16)<br>• No quantitative data of interest for our study (N=6)<br>• Neither median nor geometric mean values shown (N=3)<br>• Use of plastics for sample collection (N=2)<br>• Matrix, i.e. serum instead of urine (N=2)<br>• Data for infants (N=1)	88
TOPIC: DEHP or (ethylhexyl and phthalate) or (ethylhexylphthalate) or (ethyl and hexyl and phthalate)<br>& TOPIC: (human or dust) and concentration<br>& TOPIC: trend	16/01/20	87		15			
TOPIC: phthalate and concentration and human and urine<br>YEAR: from 2009 to 2020	28/01/20	440		93			
TOPIC: phthalate and concentration and human and urine<br>YEAR: from 2020 to 2021	22/01/21	67		16			
TOPIC: phthalate or phthalates or plasticizer or plasticizers<br>& TOPIC: human and (urine or urinary or plasma or blood or serum)<br>YEAR: from 2019 to 2020	27/04/20 and 12/10/20	391 results screened		43			
TOPIC: terephthalate or DEHT or DEHTP or DOTP or DINCH or MINCH or (cyclohexane and (dicarboxylic or dicarboxylate)) or cyclohexanedicarboxylic or cyclohexanedicarboxylate or (adipate and plasticizer)<br>& TOPIC: human and (urine or urinary or plasma or blood or serum)	05/10/20	245 results since 2009		25			
Other sources (mainly citations)	2020			48			26
TOPIC: phthalate* or terephthalate* or deht* or dotp or dinch or (cyclohexanedicarboxylic or (cyclohexane and dicarboxylate)) or plasticizer*	03/06/21[c]	434 results, of which 224 results since		9		At this stage of our study, all exclusion criteria were considered and priority data were compiled [c]	9

AND TOPIC: urin*<br>YEAR: 2020 to 2021		Sep. 2020 were screened					
Total				256	188	65	123

[a] Title, abstract, and, if needed, material and methods were screened

[b] In the table, we show the number of publications excluded based on these criteria. Moreover, for publications included in data compilation, some groups of subjects were also excluded based on the same criteria (e.g. if obese and control groups were analyzed separately, we only compiled data for the control group)

[c] At this stage (in June 2021), all exclusion criteria were considered during the screening of results. Additionally, in this last research data which were not a priority for our study (e.g. data which were not reported unadjusted, or data for non-priority age groups for our study such as seniors or younger children) were not included in the database. At this stage, priority data included relevant data in Asian and North American countries, and recent data (since 2015) in other continents.

Table S 2: list of abbreviation/s and definition/s of the parent substances and their metabolite/s found in the literature for phthalates and replacing plasticizers

**Parent substance**		**Metabolite**	
**Abbreviation/s**	**Definition/s**	**Abbreviation/s**	**Definition/s**
**Low molecular weight phthalates**			
**DMP**	Dimethyl phthalate	**MMP**	Mono-methyl phthalate
**DEP**	Diethyl phthalate	**MEP**	Mono-ethyl phthalate
**DiPrP**; DIPrP	Diisopropyl phthalate	**MiPrP**	Mono-isopropyl phthalate
**DnPrP**; DPrP	Dipropyl phthalate; Di-n-propyl phthalate	**MnPrP**	Mono-n-propyl phthalate
**DiBP**; DIBP	Di-isobutyl phthalate	**MiBP**	Mono-isobutyl phthalate; Mono-2-isobutyl phthalate
		**OH-MiBP**; 2OH-MiBP; MHiBP	2OH-Mono-isobutylphthalate; mono-hydroxy-iso-butyl phthalate; mono-2-methyl-2-hydroxypropyl phthalate.
**DnBP**; DBP	Dibutyl phthalate; di-n-butyl phthalate	[a] **MnBP**; MBP	Mono-n-butyl phthalate; monobutyl phthalate
		**OH-MnBP**; 3OH-MnBP; MHBP	3OH-Mono-n-butyl phthalate; mono-hydroxy-n-butyl phthalate; Mono(4-hydroxybutyl) phthalate (1)
**BBP**; BBzP	Benzyl butyl phthalate; butyl benzyl phthalate	**MBzP**	Mono-benzyl phthalate
**DCHP**; DcHP	Dicyclohexyl phtalate; 1,2-Benzenedicarboxylic acid, 1,2-dicyclohexyl ester	**MCHP**; McHP	Mono-cyclohexyl phtalate
**DiPeP**; DiPP	Di-isopentyl phthalate	**MiPeP**; MiPP	Mono-isopentyl phthalate
**DnPeP**; DnPP; DPeP	Di-n-pentylphthalate; Dipentyl phthalate	**MHPeP**	Mono-4-hydroxypentyl phthalate
		**MnPeP**; MPeP	Mono-n-pentyl phthalate
**DHxP**; DHP; DnHP	Dihexyl phthalate; 1,2-Benzenedicarboxylic acid, 1,2-dihexyl ester; Di-n-hexyl phthalate	**MHxP**; MnHP	Monohexyl phthalate; Mono-n-hexyl phthalate
		**OH-MHxP**	Mono-(5-hydroxyhexyl) phthalate
		**cx-MPeP**	Mono-(5-carboxypentyl) phthalate
**High molecular weight phthalates**			
**DEHP**	Di(2-ethylhexyl) phthalate; bis(2-ethylhexyl) phthalate	**5cx-MEPP**; MECPP; cx-MEPP	Mono(2-ethyl-5-carboxy-pentyl) phthalate; Mono-ethyl-carboxy-pentyl phthalate
		**5OH-MEHP**; MEHHP; OH-MEHP	Mono(2-ethyl-5-hydroxy-hexyl) phthalate; Mono-ethyl-hydroxy-hexyl phthalate
		**5oxo-MEHP**; MEOHP; oxo-MEHP	Mono(2-ethyl-5-oxo-hexyl) phthalate; Mono-ethyl-oxo-hexyl phthalate
		**MCMHP**; 2cx-MMHP	Mono-carboxy-methyl-hexyl phthalate; Mono-2-carboxymethyl-hexyl phthalate
		**MEHP**	Mono(2-ethylhexyl) phthalate; Mono-ethyl-hexyl phthalate
**DiHpP**	Diisoheptyl phthalate	**MiHpP**	Monoisoheptyl phthalate
		**MOHpP**	Mono-oxoheptylphthalate
		**cx-MHxP**; MCHxP	Mono-(6-carboxyhexyl) phthalate; Monocarboxyhexyl phthalate
**DnHpP**; DHpP	Di-n-heptyl phthalate	**MHHpP**; OH-MHpP	Monohydroxyheptyl phthalate; Mono-(6-hydroxyheptyl) phthalate
		**MnHpP**; MHpP	Mono-n-heptyl phthalate; Monoheptyl phthalate; mono-2-heptyl phthalate
**DOP**; DnOP		**MOP**; MnOP	Mono-n-octyl phtalate; Monooctyl phthalate

	Dioctyl phthalate; 1,2-Benzenedicarboxylic acid, 1,2-dioctyl ester; Di-n-octyl phtalate	**MCHpP**; mCHpP	Mono(7-carboxyheptyl) phthalate; Mono-carboxy-n-heptyl phthalate
[b] **DPHP**	Bis(2-propylheptyl) phthalate; 1,2-Benzenedicarboxylic acid, 1,2-bis(2-propylheptyl) ester	**cx-MPHP**; cx-MPHxP	mono (2-propyl-6-carboxyhexyl)-phthalate; mono (propyl-6-carboxyhexyl)-phthalate
		**OH-MPHP**	mono(2-propyl-6-hydroxyheptyl)-phthalate; mono(propyl-6-hydroxyheptyl)-phthalate; 6-Hydroxy Monopropylheptylphthalate;
		**oxo-MPHP**	mono (2-propyl-6-oxo-heptyl)-phthalate; mono (propyl-6-oxo-heptyl)-phthalate
		**MPHP**	monopropylpheptyl phthalate; mono(2-propylheptyl) phthalate
**DINP**; DiNP	Diisononyl phtalate; 1,2-Benzenedicarboxylic acid, 1,2-diisononyl ester	**cx-MINP**; cx-MiNP; MCiOP; MCOP	Mono-carboxy-octyl phthalate; Mono-carboxy-isooctyl phthalate; Mono(6-carboxy-2-methylheptyl) phthalate (2-4); 7-Carboxy-(mono-methyl-heptyl) phthalate (5, 6); Mono-(4-methyl-7-carboxyheptyl)phthalate (7)
		**MINP**; MiNP; MNP	Mono-isononyl phthalate
		**OH-MINP**; OH-MiNP; MHiNP	Mono-hydroxy-isononyl phthalate (3, 4); 7-OH-(Mono-methyl-octyl) phthalate (5)
		**oxo-MINP**; oxo-MiNP; MOiNP; MONP	Mono-oxo-isononyl phthalate (3, 4); 7-Oxo-(Mono-methyl-octyl) phthalate (5)
[b] **DIDP**; DiDP	Diisodecyl phthalate; 1,2-Benzenedicarboxylic acid, 1,2-diisodecyl ester	**MIDP**; MiDP	Mono-isodecyl phthalate (3, 8); Mono(8-methyl-1-nonyl) phthalate (9)
		**cx-MIDP**; cx-MiDP; MCNP; MCINP; MCiNP	Mono(2,7-methyl-7-carboxy-heptyl) phthalate (5); mono-carboxy-isononyl phthalate; Monocarboxynonyl phthalate (2, 10); Mono-(9-carboxynonyl) phthalate (3)
		**OH-MIDP**; OH-MiDP; MHiDP	Mono-hydroxy-isodecyl phthalate (10); 6-OH-mono-propylheptyl phthalate; 6-hydroxypropylheptyl phthalate (5, 11); Mono-(9-hydroxydecyl) phthalate (3)
		**oxo-MIDP**; oxo-MiDP; MOiDP	Mono-oxo-isodecyl phthalate (included under this definition as a DIDP metabolite by Health Canada and Schutze et al. (4, 10); 6-oxo-mono-propylheptyl phthalate (5); Mono-(9-oxodecyl) phthalate (3);
Several parental phthalates (including DnBP, DnPeP, DnOP, DiNP, DiDP; (5, 12)		**MCPP**; 3cx-MPP	Mono-3-carboxypropyl phtalate; 3-carboxyl-mono-propyl phthalate
**Phthalate replacements (substitutes)**			
**DINCH** (Hexamoll® DINCH)	1,2-Cyclohexanedicarboxylic acid, 1,2-diisononyl ester; di-(iso-nonyl)-cyclohexane-1,2-dicarboxylate;	**cx-MINCH**; MCOCH	Cyclohexane-1,2-dicarboxylic acid-mono (carboxy-isooctyl) ester
		**MINCH**	cyclohexane-1,2-dicarboxylic acid-mono(isononyl) ester
		**OH-MINCH**; MHINCH; MHNCH	Cyclohexane-1,2-dicarboxylic acid-mono (hydroxyl-iso-nonyl) ester (13-15)
		**oxo-MINCH**; MOiNCH; MONCH	Cyclohexane-1,2-dicarboxylic acid-mono (oxo-isononyl) ester (13-15)
		CHDA	Cyclohexane-1,2-dicarboxylic acid
**DEHT**; DEHTP; DOTP	Bis(2-ethylhexyl) terephthalate; di(2-ethylhexyl) terephthalate;	**2cx-MMHTP**	1-mono-(2-carboxyl-methyl-hexyl) benzene-1,4-dicarboxylate; Mono-(2-carboxyl-methyl-hexyl) terephthalate

	1,4-Benzenedicarboxylic acid, 1,4-bis(2-ethylhexyl) ester; Dioctyl terephthalate	**5cx-MEPTP**; MECPTP	Mono-2-ethyl-5-carboxypentyl terephthalate; 1-mono-(2-ethyl-5-carboxyl-pentyl) benzene-1,4-dicarboxylate;
		**5OH-MEHTP**; MEHHTP	Mono-2-ethyl-5-hydroxyhexyl terephthalate; 1-Mono-(2-ethyl-5-hydroxy-hexyl) benzene-1,4-dicarboxylate;
		**5oxo-MEHTP**	1-mono-(2-ethyl-5-oxo-hexyl) benzene-1,4-dicarboxylate; Mono-(2-ethyl-5-oxo-hexyl) terephthalate
		**MEHTP**	Mono(2-ethylhexyl) terephthalate
[c] **DEHA**	Di(2-ethylhexyl)adipate; Di-octyl adipate	**MEHA**	Mono-2-ethylhexyl adipate
		**MEHHA**	Mono-2-ethylhydroxyhexyl adipate
		**MEOHA; oxo-MEHA**	Mono(2-ethyl-5-oxohexyl) adipate
[c] **DnBA**	Di-n-butyl adipate; Hexanedioic acid, dibutyl ester	**3cx-MnPrA**	3-carboxy-mono-n-propyl adipate
		**3OH-MnBA**	3-hydroxy-mono-n-butyl adipate
		**MnBA**	mono-n-butyl adipate
[c] **TCEPho**	Tris(2-chloroethyl)phosphate	**DCEPho**; BCEPho	Di(2-chloroethyl)phosphate; bis(chloroethyl) phosphate; bis(2-chloroethyl) phosphate
[c] **TnBPho**	Tri-n-butyl phosphate	**DnBPho**; DBPho	Di-n-butyl phosphate; dibutyl phosphate
[c] **TCPho**	Tricresyl phosphate-isomers	**DmCPho**	Di-m-cresyl phosphate
		**DoCPho**	Di-o-cresyl phosphate
		**DpCPho**	Di-p-cresyl phosphate

In the table, references are given for definitions that were relatively heterogeneous between publications

[a] MnBP can also be formed from BBP (16)

[b] DIDP and DPHP metabolites may not be clearly distinguished through high performance liquid chromatography coupled to tandem mass spectrometry (HPLC–MS/MS) (5). For these substances we used the same classification (i.e. classification as either DIDP or DPHP metabolites) that was shown in each publication

[c] Our literature research did not focus on adipates or phosphates, for which data were found under the general keyword "plasticizers". Additional data exist in the literature for these substances, notably for phosphates.

Table S 3: number of central values (priority for median, followed by geometric mean) of unadjusted phthalate urinary concentrations per metabolite and country/region in adults, included in the "PhthaLit" database

Measured.subst.abbreviation	China	India	Israel	Japan	Korea	Malaysia	Saudi Arabia	Taiwan	Vietnam	Austria	Belgium	Czech Republic	Denmark	Finland	France	Germany	Hungary	Italy	Norway	Slovakia	Slovenia	Spain	Sweden	United Kingdom	Canada	US	Australia	All countries
MMP	18	1	0	1	2	1	2	3	1	0	1	1	3	0	1	4	1	0	1	2	0	0	0	0	3	2	1	49
MEP	19	1	0	1	2	1	2	3	1	1	3	2	5	1	1	5	1	2	3	3	1	1	2	1	3	4	1	77
MiBP	18	1	1	1	3	1	2	3	1	1	3	1	5	1	1	5	0	0	3	1	1	1	0	1	3	4	1	70
MnBP	19	1	1	1	6	1	2	3	1	1	3	1	5	1	1	5	0	2	3	2	1	1	1	1	3	4	1	78
OH-MnBP	0	0	0	0	0	0	0	0	0	0	0	0	3	0	0	4	0	0	0	0	0	0	0	0	1	2	0	10
MBzP	17	1	1	1	5	1	2	3	1	1	3	2	5	1	1	5	1	2	3	1	1	1	2	1	3	4	1	77
MCHP	6	1	0	1	3	1	2	0	1	1	0	1	3	1	1	4	1	0	0	2	0	0	0	0	3	1	1	34
MnPeP	1	0	0	0	2	0	1	0	0	1	0	0	3	0	0	4	0	0	0	0	0	0	0	0	0	0	0	12
5cx-MEPP	12	1	1	1	4	1	2	3	1	1	1	1	4	1	0	4	0	0	2	1	0	0	2	0	2	11	1	57
5OH-MEHP	19	1	1	1	9	1	2	3	1	1	3	2	4	1	1	4	1	2	3	2	1	1	2	1	3	8	1	79
5oxo-MEHP	19	1	1	1	9	1	2	3	1	1	3	2	4	1	1	4	1	0	3	2	1	1	2	1	3	4	1	73
MCMHP	8	1	1	1	2	1	2	2	1	0	0	0	3	0	0	0	0	0	1	0	0	0	0	0	1	0	0	24
MEHP	19	1	1	1	6	1	2	3	1	1	3	2	4	1	1	4	1	2	3	3	1	1	2	1	3	4	2	74
MOP	4	1	0	1	2	1	2	0	1	1	0	0	4	1	1	4	0	0	0	1	0	0	0	0	3	1	3	31
cx-MINP	0	0	1	0	2	0	0	0	0	0	0	1	4	1	0	4	0	0	2	0	0	1	2	0	1	7	1	27
MINP	7	1	0	1	2	1	2	3	1	1	0	0	4	1	1	0	0	0	0	2	0	0	0	0	3	4	2	36
OH-MINP	0	0	1	0	1	0	0	0	0	0	0	1	4	0	0	4	0	0	2	0	0	0	2	0	1	0	0	16
oxo-MINP	0	0	1	0	0	0	0	0	0	0	0	1	4	0	0	4	0	0	2	0	0	0	2	0	1	1	0	16
cx-MIDP	0	0	0	0	1	0	0	0	0	0	0	0	3	1	0	4	0	0	0	0	0	0	0	0	1	4	1	15
MIDP	0	0	0	0	2	0	2	1	0	1	0	0	4	0	0	0	0	0	0	0	0	0	0	0	1	0	3	14
MCPP	7	1	0	1	3	1	2	0	1	1	0	1	4	0	1	4	0	0	0	0	0	0	0	0	3	4	1	35
cx-MINCH	0	0	0	0	1	0	0	0	0	0	0	0	3	0	0	7	0	0	1	0	0	0	0	0	0	4	0	16
OH-MINCH	0	0	0	0	1	0	0	0	0	0	0	0	3	0	0	7	0	0	2	0	0	0	0	0	1	6	0	20
oxo-MINCH	0	0	0	0	0	0	0	0	0	0	0	0	0	0	0	7	0	0	1	0	0	0	0	0	1	3	0	12

5cx-MEPTP	0	0	0	0	0	0	0	0	0	0	0	0	3	0	0	6	0	0	0	0	0	0	0	0	0	5	0	14
5OH-MEHTP	0	0	0	0	1	0	0	0	0	0	0	0	3	0	0	6	0	0	0	0	0	0	0	0	0	5	0	15
All metabolites	198	14	11	14	82	14	36	33	14	14	23	22	133	14	12	153	7	10	37	22	7	8	19	7	50	101	22	1105[a]

Main matrix (white and blue scale): only countries for which the number of central values (n) was >= 5, and metabolites for which n was >= 10 are shown

Totals (white and grey scale): including all countries and metabolites in the database

The abbreviations of all metabolites are defined in Table S 2

a More data were compiled later. The final version of the “PhthaLit” database, used in the analyses shown in the main manuscript, contains n = 1208 unadjusted central values for adults

Table S 4: number of central values (priority for median, followed by geometric mean) of unadjusted phthalate urinary concentrations per metabolite and country/region in children, included in the "PhthaLit" database

Measured.subst.abbreviation	China	Egypt	Korea	Saudi Arabia	Taiwan	Austria	Belgium	Czech Republic	Denmark	France	Germany	Greece	Hungary	Norway	Poland	Portugal	Slovakia	Slovenia	Spain	Sweden	United Kingdom	Canada	US	Mexico	Brazil	All countries
MMP	12	0	0	1	6	0	0	1	0	1	2	0	1	0	1	1	2	0	0	0	0	2	2	0	1	33
MEP	14	2	0	1	6	2	2	1	3	1	4	1	1	1	2	2	2	1	1	2	1	2	5	3	1	66
MiBP	10	2	0	1	5	2	2	2	3	1	4	1	0	1	2	2	1	1	1	0	1	2	5	3	1	58
MnBP	14	2	1	1	6	2	2	2	3	1	4	1	0	1	2	2	1	1	1	1	1	2	5	3	1	65
MBzP	9	2	1	1	6	2	2	3	3	1	4	1	1	1	2	2	1	1	1	2	1	2	5	3	1	63
MCHP	5	0	0	1	0	2	0	1	0	1	2	0	1	0	1	1	2	0	0	0	0	2	1	0	1	21
5cx-MEPP	8	2	1	1	2	2	0	0	2	0	2	0	0	1	1	1	1	0	0	2	0	1	5	3	1	36
5OH-MEHP	14	2	1	1	6	2	2	3	2	1	3	1	1	1	1	1	2	1	1	2	1	2	5	3	1	62
5oxo-MEHP	14	2	1	1	6	2	2	3	2	1	3	1	1	1	1	1	2	1	1	2	1	2	5	3	1	62
MEHP	14	2	0	1	6	2	2	3	2	1	2	1	1	1	1	1	2	1	1	2	1	2	5	3	1	60
MOP	4	0	0	1	0	2	0	0	1	1	2	0	0	0	1	1	1	0	0	0	0	2	1	0	1	18
cx-MINP	1	2	1	0	0	0	0	0	1	0	2	0	0	1	1	1	0	0	1	2	0	1	5	0	1	20
MINP	3	0	0	1	4	2	0	0	1	1	0	0	0	0	0	0	1	0	0	0	0	2	5	0	1	21
oxo-MINP	0	0	0	0	0	0	0	0	1	0	3	0	0	1	1	1	0	0	0	2	0	1	2	0	0	12
cx-MIDP	0	2	1	0	0	0	0	0	0	0	2	0	0	0	1	1	0	0	0	1	0	1	5	0	1	15
MCPP	1	2	1	1	0	2	0	0	1	1	2	0	0	0	1	1	0	0	0	0	0	2	5	3	1	24
All metabolites	134	22	8	18	53	28	14	19	27	12	72	7	7	11	25	29	18	7	8	23	7	37	86	27	24	750[a]

Main matrix (white and blue scale): only countries for which the number of central values (n) was >= 5, and metabolites for which n was >= 10 are shown

Totals (white and grey scale): including all countries and metabolites in the database

The abbreviations of all metabolites are defined in Table S 2

a More data were compiled later. The final version of the "PhthaLit" database, used in the analyses shown in the main manuscript, contains n = 859 unadjusted central values for children

Table S 5: studies that shared a given Study ID number in the database (data for non-European countries)

Study.ID.number	Study.name	Country / region	Specific location	Biomonitoring plan / biobank	Sampling years	Average year	Descript.Population	Age.group.category	N	Gender	Matrix
6	Choi et al., 2017 (26); Park et al., 2019 (27)	South Korea	Several locations in South Korea	Korean National Environmental Health Survey (KoNEHS)	June 2012 - May 2014	2013	Adults (> 19 y; N=6470)	adults	6470	Both	Spot urine
	Park et al., 2016 (28)				2009-2011	2010	Adults (> 19 y; N=6274)	adults	6274		
	Lim S., 2020 (29)				Aug. 2015 - Jul. 2017	2016	Children (6-11 y; N=839)	children	839		
							Teenagers (12-17 y; N=807)	teenagers	807		
							Adults (19-86 y; N=3759)	adults	3759		
29	Lee I. et al., 2019 (23); Kang et al., 2019 (30)	South Korea	Several locations	-	2015–2016	2016	Women (N=459 in Lee et al., 2019; N=441 in Kang et al., 2019; same population in both studies; 20–48 years-old)	adults	450	Female	Spot urine
20	Chen JS et al., 2019 (31)	China	Shanghai, 9 urban and suburban districts/counties	Shanghai Food Consumption Survey (SHFCS)	2012	2012	Adults (N=1663)	adults	1663	Both	Spot urine
	Dong et al., 2018 (32)				Sep. 2012 - Aug. 2014	2013	Boys (8-11 y; N=64)	children	64	Male	
							Girls (8-11 y; N=65)	children	65	Female	
							Teenage boys (12-19 y; N=76)	teenagers	76	Male	
							Teenage girls (12-19 y; N=66)	teenagers	66	Female	
							Men (45-60 y; N=471)	adults	471	Male	
							Women (45-60 y; N=401)	adults	401	Female	
16	Liao et al., 2017, 2018 (33, 34)	China	Shanghai, several areas	China, Children, Homes, Health (CCHH) study	2013-2014	2014	Children (5-10 y; N=434)	children	434	Both	Morning urine

93	Yao et al., 2019, 2020 (24, 35)	China	Shenzhen, eight districts: Guangming, Baoan, Dapeng, Futian, Nanshan, Longhua, Longgang and Pingshan	EHC-PRD study (environment and health in children in the Pearl River Delta, China)	Sep 2016 - Jun 2017	2017	Children (6–8 y, average age 7.8 y; N=1490; 59% boys)	children	1490	Both	Spot urine
97	Huang et al., 2021 (36)	Taiwan	11 cities	Taiwan Environmental Survey for Toxicants (TEST) and National and Nutrition Health Survey in Taiwan (NAHSIT)	May-Dec 2013	2013	Adults (52.7 ± 17.5 y; N=217)	adults	217	Both	Spot urine
	Liao et al., 2021 (37)		20 cities, different areas and regions		May 2013 - Dec 2016	2015	Children (7-11 y; N=336)	children	336	Both	Morning urine
							Adults (>= 18 y; N=1264)	adults	1264	Both	
73	Goodrich et al., 2016 (38)	Mexico	Mexico city	Early Life Exposures in Mexico to Environmental Toxicants (ELEMENT) birth cohorts	2011-2012	2012	Children and teenagers (8-14 y; N=238)	children	238	Both	Spot urine
	Lewis et al., 2013 (39)				2010	2010	Boys (8-13 y; N=53)	children	53	Male	Spot urine
78	Feng et al., 2020 (40)	Canada	Montreal	Protocol number A01-M14-10B	2009 to 2012	2011	Men (fertile, control) (18-39 y; N=106)	adults	106	Male	Spot urine
	Albert et al., 2018 (41)						Men (fertile, control) (18-41 y; N=117)	adults	117		
35	Tang et al., 2020 (42)	Australia	Queensland	-	Nov 2012-Dec 2013	2013	General population (N=24 pools of 100 individual samples each, 8 pools for average ages < 18 y and 16 pools for average ages > 18 y)	adults	2400	Both	Spot urine
	Gomez-Ramos et al., 2016 (43)					2013					

Table S 6: studies that shared a given Study ID number in the database (data for European countries)

Study.ID	Study.name	Country / region	Specific location	Biomonitoring plan / biobank	Sampling years	Average year	Descript.Population	Age.group.category	N	Gender	Matrix
5	Schoeters et al., 2017 (44); Koppen et al., 2020 (45)	Belgium	Flanders	FlemishEnvironment and Health Study (FLEHS)	2013	2013	Adolescents (14-17 y; N=207 in 2013, FLEHS III)	teenagers	207	Both	Spot urine
					2017-2018	2018	Adolescents (14.77±0.47 y; N=416)	teenagers	416		
2	Schwedler et al., 2020 (13)	Germany	Several	German Environmental Survey of Children and Adolescents (GerES)	Jan 2015 - Jun 2017	2016	Children (6-10 y; N=166)	children	166	Both	Morning urine
	Schwedler et al., 2020 (12)						Children (6-10 y; N=736)	children	736		
	Schwedler et al., 2020. (46)						Children (6-10 y; N=696)	children	696		
3	Schutze et al., 2015 (10)	Germany	Münster	German Environmental Specimen Bank-German Environment Agency (ESB-UBA)	Several campaigns	Average year per campaign	Young adults (university students; 20 to 30 year-old, N=60 per sampling year)	adults	60	Both	24-h urine
	Schmidtkunz et al., 2019 (47)										
	Lessmann et al., 2019 (48)										
	Schutze et al., 2014 (49)										
	Kasper-Sonnenberg et al., 2019 (14)										
	Koch et al., 2017 (5)										
8	Fromme et al., 2013, 2015 (50, 51)	Germany	Bavaria, Berlin, North Rhine-Westfalia	Länderuntersuchungsprogramme, LUPE 3	Nov 2011-May 2012	2012	Children (1.7 to 6.7 y; N=208-663)	children	208-663	Both	Spot urine
	Fromme et al., 2014 (52)						Children (1.8-6.7 y; N=312)		312		

39	Den Hond et al., 2015 (53)	Several		Consortium to Perform Human Biomonitoring on a EuropeanScale (COPHES) and Demonstration of a study to Coordinate and Perform Human Biomonitoringon a European Scale (DEMOCOPHES)	2011 - 2012 (exact dates slightly different per country)	2011 or 2012	Children (5-11 years-old; N=120 approx.) and Mothers (N=120 approx.)	children / adults	Dependent on the age group and country	Both (children) / Female (mothers)	Morning urine
	Koppen et al., 2019 (54)	Belgium				2012					
	Cerna et al., 2015 (55)	Czech Republic				2012					
	Schwedler et al., 2017 (56)	Germany				2011					
	Cullen et al., 2017 (57)	Ireland				2012					
	Runkel et al., 2020 (58)	Slovenia			2011	2011					
	Cutanda et al., 2015 (6)	Spain			Oct. 2011 - Jan. 2012	2012					
	Larsson et al., 2014 (59)	Sweden			Sep. 2011 - Feb. 2012	2012					
	Exley et al., 2015 (60)	United Kingdom			Jan to Apr. 2012	2012					
40	Myridakis et al., 2015 (61)	Greece	Crete	Rhea cohort	March 2009–June 2011_Rhea project	2010	Toddlers (2.3 ± 0.72 y; N=390)	younger children	390	Both	Spot urine
	Myridakis et al., 2016 (62)				2011-2013	2012	Children (4.24 ± 0.24 y; N=500)	children	500		
	Haug et al., 2018 (63)			Early-Life Exposome project (HELIX), Rhea cohort	2014	2014	Children (6.5±0.3 y; N=199)	children	199		Morning urine
		Lithuania		Early-Life Exposome project (HELIX)	2014	2014	Children (6.5±0.5 y; N=204)	children	204		
48	Tranfo et al., 2013 (64)	Italy	Lazio	-	2011	2011	Women (29-47 y; N=83)	adults	83	Female	Spot urine
	Tranfo et al., 2018 (65)		Central Italy		2016	2016	Women (N=111 in 2016)	adults	111		

50	Giovanoulis et al., 2016 (66); Alves et al., 2017 (67)	Norway	Oslo	Advanced Tools for Exposure Assessment and Biomonitoring (A-TEAM) project	Winter 2013-2014_A-TEAM project	2014	Adults (20-66 y; N=61)	adults	61	Both	Spot urine
57	Milošević et al., 2018 (68)	Serbia	Vojvodina region	-	June 2015 to June 2016	2016	Men, normal-weight (18-55 y; N=51)	adults	51	Male	Morning urine
	Milošević et al., 2020 (69)				n.s.	-	Adults, normal-weight (18-50 y; N=100)	adults	100	Both	Morning urine
52	Polanska et al., 2014 (70)	Poland	Łódź	Polish Mother and Child Cohort Study (REPRO_PL)	Unclear, 2010 – 2013 approx.	-	Children (23–28 months; N=165)	younger children	165	Both	Spot urine
	Garí et al., 2019 (71)				2014-2015	2015	Children (7.2±0.23 y; N=250)	children	250		
53	Correia-Sá et al., 2017 (72)	Portugal	Oporto and Aveiro	-	2014-2015	2015	Children and teenagers, normal-weight or underweight (4-18 y; N=39-43)	children	39-43	Both	Morning urine
	Correia-Sá et al., 2018 (73)										
	Lessmann et al., 2017 (74)										

S2. Supplementary Figures

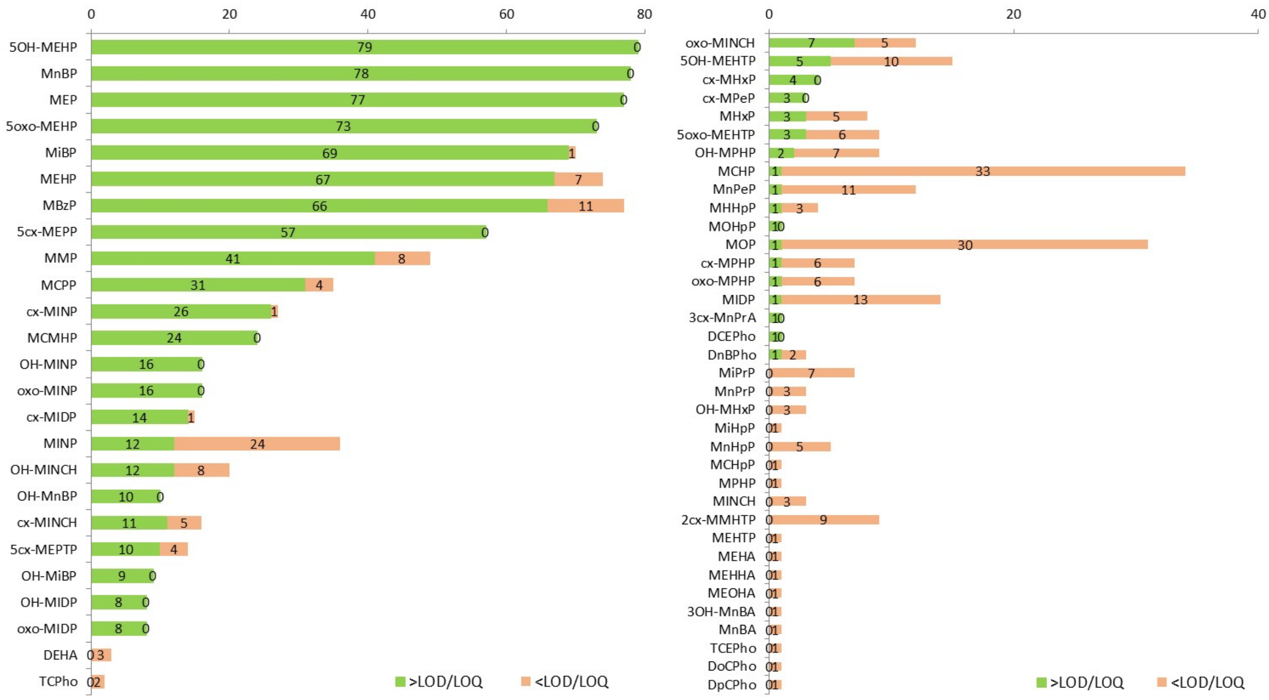

Figure S 1. Number of measured (>LOD/LOQ, green) and censored (<LOD/LOQ, orange) central values per measured substance in adults, compiled in the "PhthaLit" database

LOD: limit of detection; LOQ: limit of quantification.
The metabolites in this Figure are organized from higher to lower number of measured data per substance.
The abbreviations of all metabolites are defined in Table S 2.

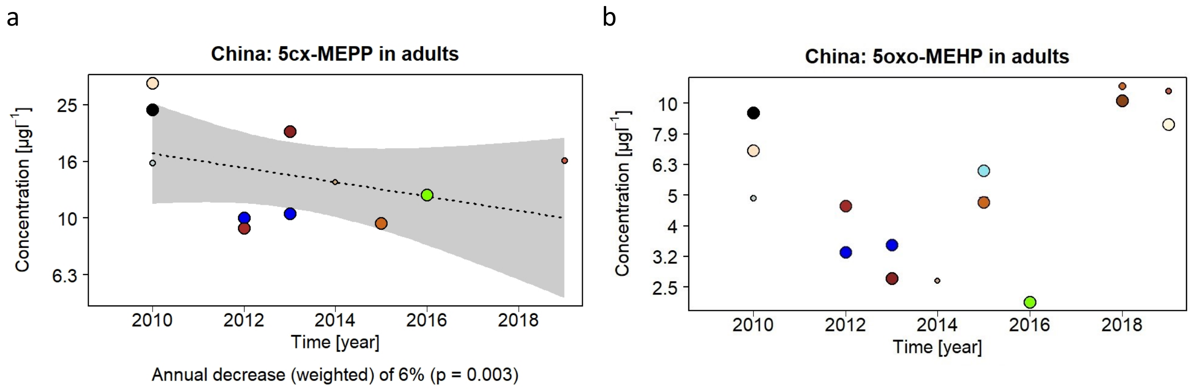

Figure S 2: time-trend in the urinary concentrations of 5cx-MEPP (DEHP metabolite, µg/L) in Chinese adults (panel a); available data for 5oxo-MEHP (DEHP metabolite) in Chinese adults (panel b)

Each symbol is a central value (i.e. median or geometric mean). The size of the symbols is related to the study-specific sample size (N) and its weight in the time-trend calculation (see Statistical methods, time-trend analysis). Data from the same data source (Study ID number, see Data traceability in the "PhthaLit" database) are identified by symbols with the same colour in all panels and Figures. Grey area: 90% confidence interval.

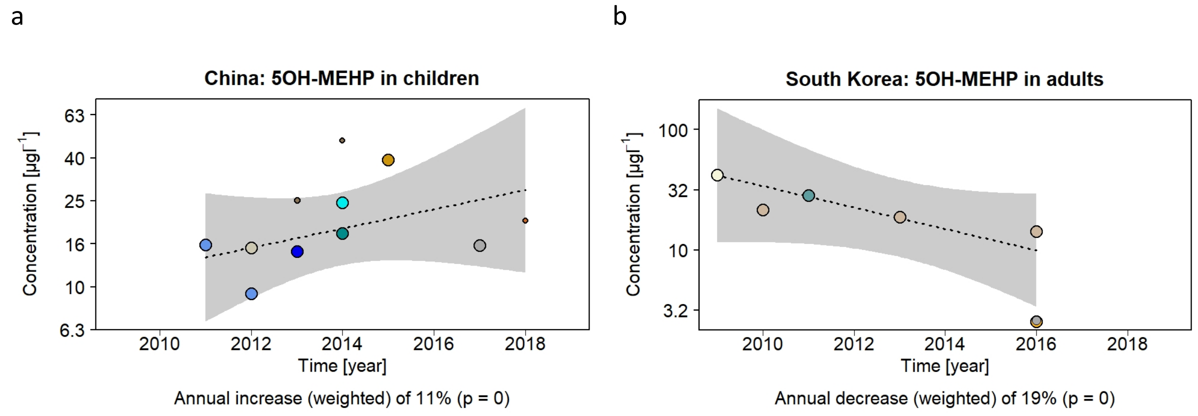

Figure S 3: time-trends in the urinary concentrations (µg/L) of the DEHP metabolite 5OH-MEHP in Chinese children (panel a) and Korean adults (panel b)

Each symbol is a central value (i.e. median or geometric mean). The size of the symbols is related to the study-specific sample size (N) and its weight in the time-trend calculation (see Statistical methods, time-trend analysis). Data from the same data source (Study ID number, see Data traceability in the "PhthaLit" database) are identified by symbols with the same colour in all panels and Figures. Grey area: 90% confidence interval. The p-value was < 0.001 for both trends

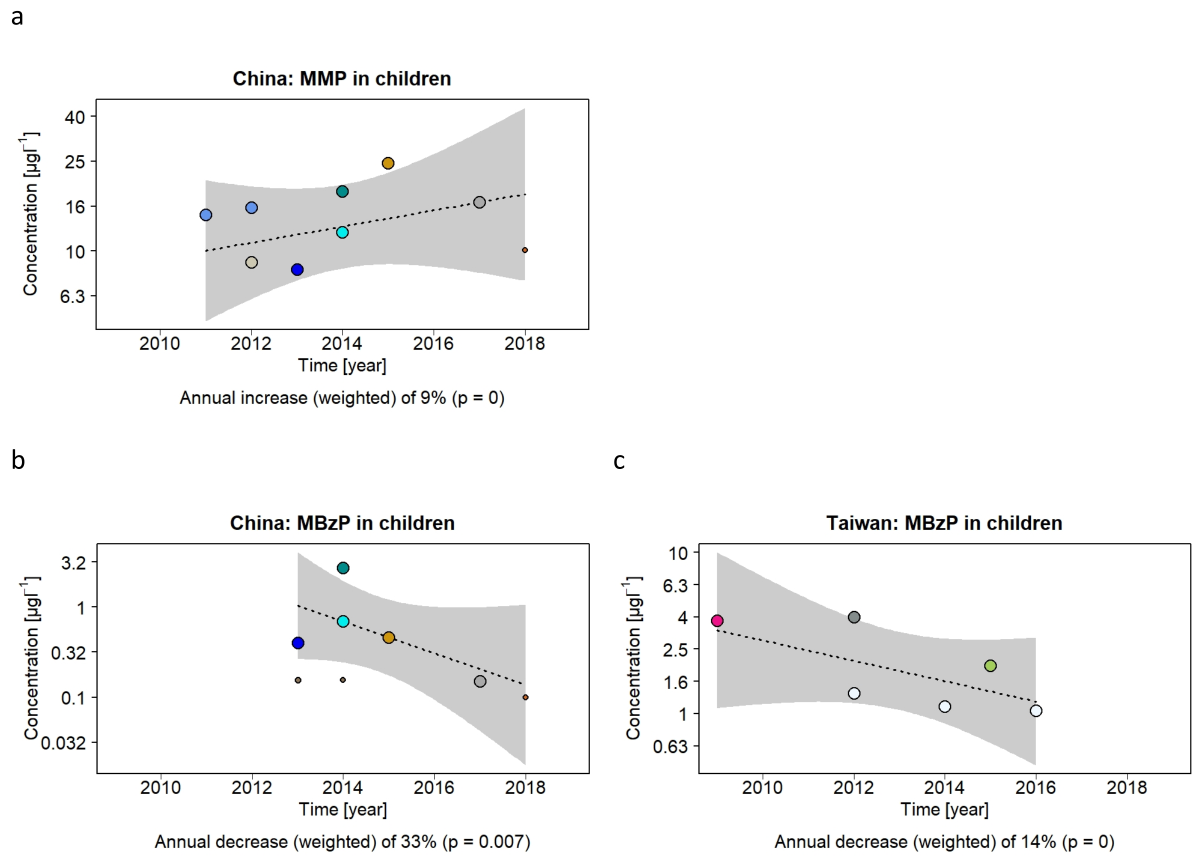

Figure S 4: time-trends in the urinary concentrations (µg/L) of: MMP (DMP metabolite) in Chinese children (panel a); MBzP (BBP metabolite) in Chinese children (panel b) and in Taiwanese children (panel c)

Each symbol is a central value (i.e. median or geometric mean). The size of the symbols is related to the study-specific sample size (N) and its weight in the time-trend calculation (see Statistical methods, time-trend analysis). Data from the same data source (Study ID number, see Data traceability in the "PhthaLit" database) are identified by symbols with the same colour in all panels and Figures. Grey area: 90% confidence interval. The p-value was < 0.001 for the trends shown in panels a and c

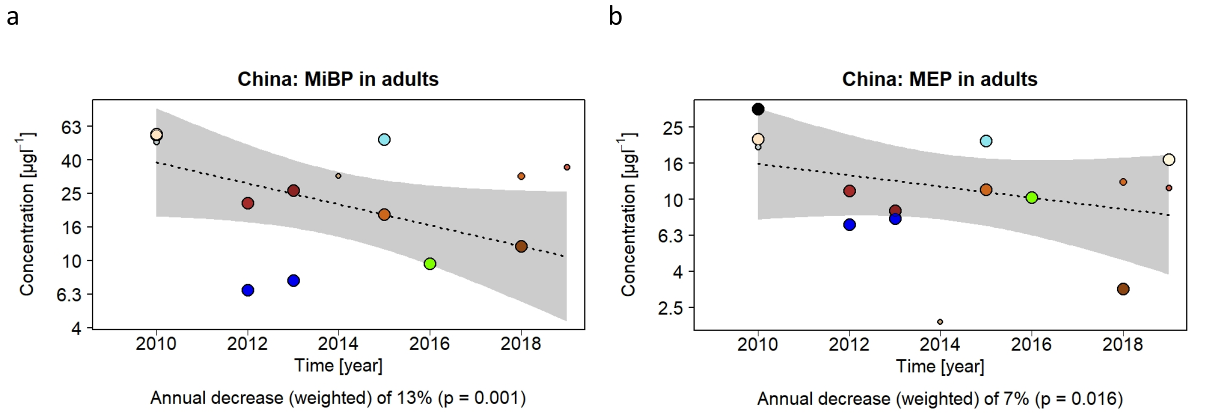

Figure S 5: time-trends in the urinary concentrations (µg/L) of MiBP (DiBP metabolite) and MEP (DEP metabolite) in Chinese adults (panels a and b, respectively)

Each symbol is a central value (i.e. median or geometric mean). The size of the symbols is related to the study-specific sample size (N) and its weight in the time-trend calculation (see Statistical methods, time-trend analysis). Data from the same data source (Study ID number, see Data traceability in the "PhthaLit" database) are identified by symbols with the same colour in all panels and Figures. Grey area: 90% confidence interval.

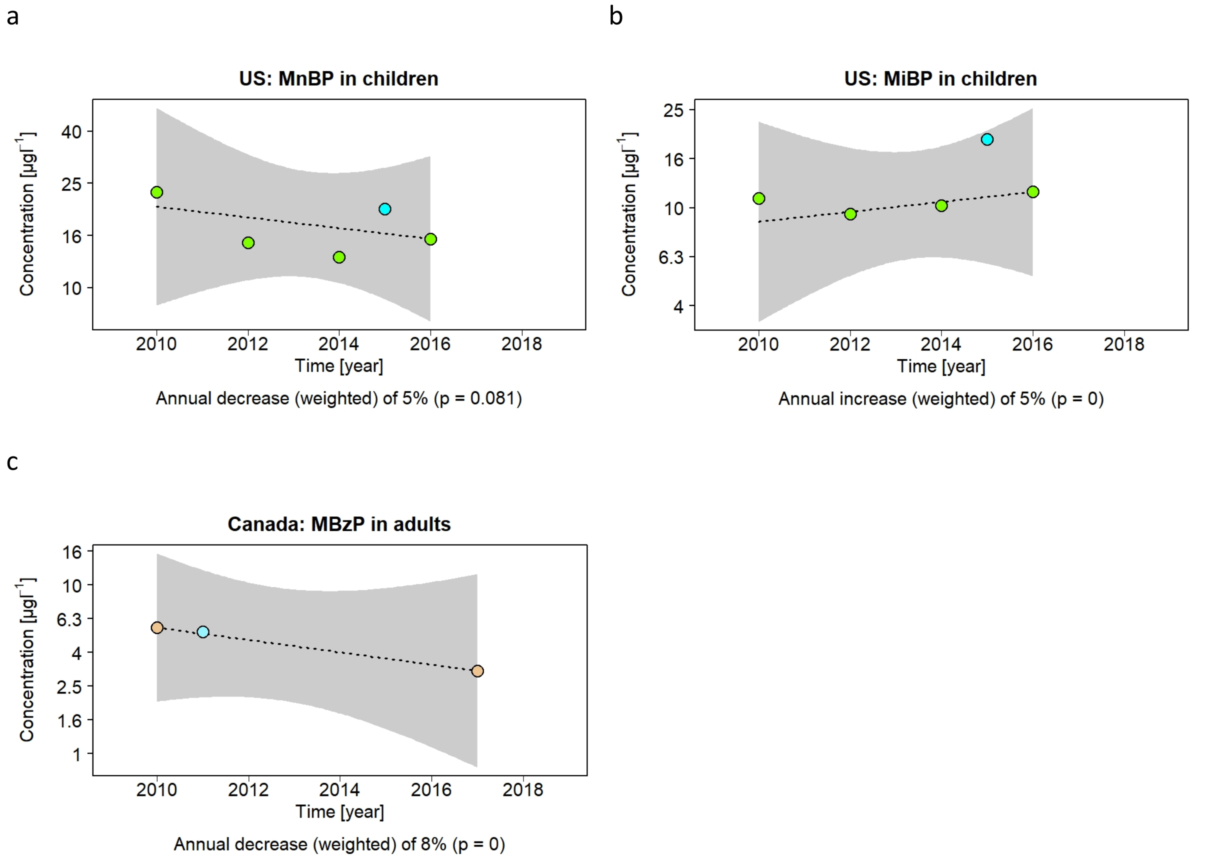

Figure S 6: time-trends in the urinary concentrations (µg/L) of: MnBP (DnBP metabolite) and MiBP (DiBP metabolite) in US children (panels a and b, respectively); MBzP (BBP metabolite) in Canadian adults (panel c)

Each symbol is a central value (i.e. median or geometric mean). The size of the symbols is related to the study-specific sample size (N) and its weight in the time-trend calculation (see Statistical methods, time-trend analysis). Data from the same data source (Study ID number, see Data traceability in the "PhthaLit" database) are identified by symbols with the same colour in all panels and Figures. Grey area: 90% confidence interval. The p-value was < 0.001 for the trends shown in panels b and c

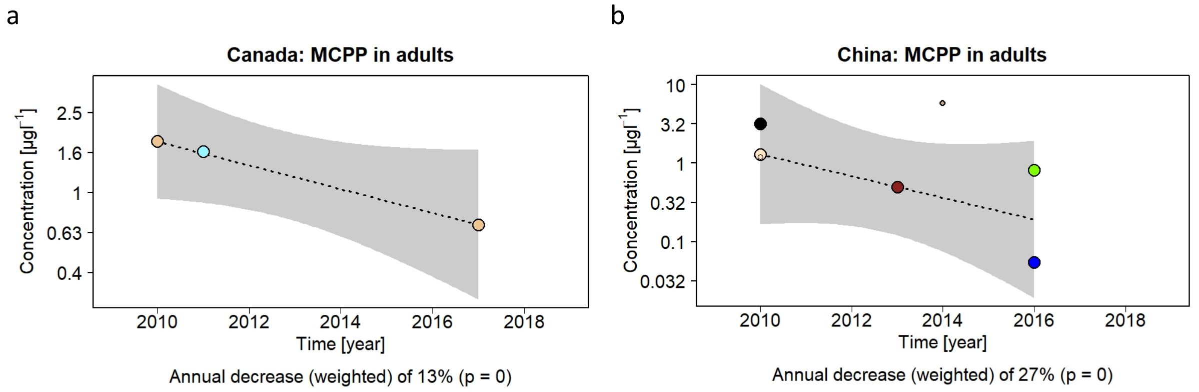

Figure S 7: time-trends in the urinary concentrations (µg/L) of MCPP in adults from Canada (panel a) and China (panel b)

Each symbol is a central value (i.e. median or geometric mean). The size of the symbols is related to the study-specific sample size (N) and its weight in the time-trend calculation (see Statistical methods, time-trend analysis). Data from the same data source (Study ID number, see Data traceability in the "PhthaLit" database) are identified by symbols with the same colour in all panels and Figures. Grey area: 90% confidence interval. The p-value was < 0.001 for the trends shown in both panels

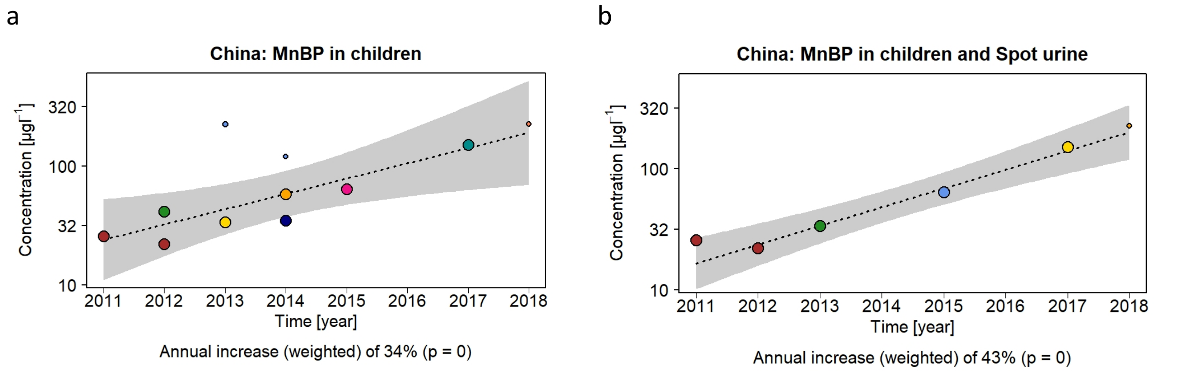

Figure S 8: time-trends for MnBP in Chinese children: a) including all data; b) per sample type (spot urine)

Each symbol is a central value (i.e. median or geometric mean). The size of the symbols is related to the study-specific sample size (N) and its weight in the time-trend calculation (see Statistical methods, time-trend analysis). Within one panel, one colour identifies one Study ID number. However, in Figures S 8 to S 13, the colours are not comparable between panels and not comparable with the colours of other Figures in the manuscript. Grey area: 90% confidence interval. The p-value was < 0.001 for the trends shown in both panels

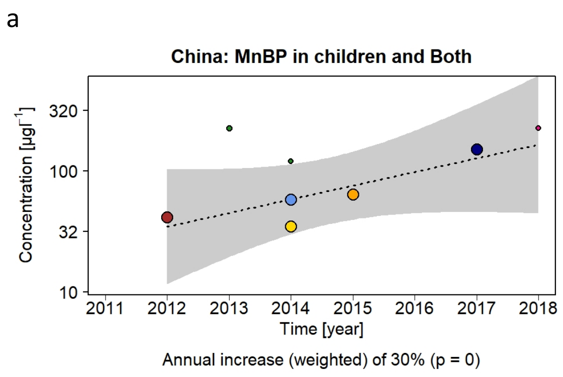

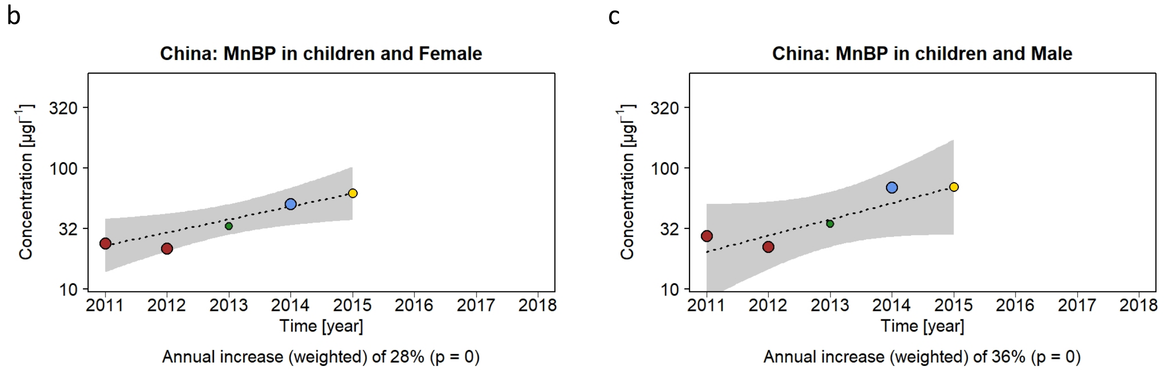

Figure S 9: significant time-trends for MnBP in Chinese children per gender (a, both genders; b, girls; c, boys)

Each symbol is a central value (i.e. median or geometric mean). The size of the symbols is related to the study-specific sample size (N) and its weight in the time-trend calculation (see Statistical methods, time-trend analysis). Within one panel, one colour identifies one Study ID number. However, in Figures S 8 to S 13, the colours are not comparable between panels and not comparable with the colours of other Figures in the manuscript. Grey area: 90% confidence interval. The p-value was < 0.001 for the three trends

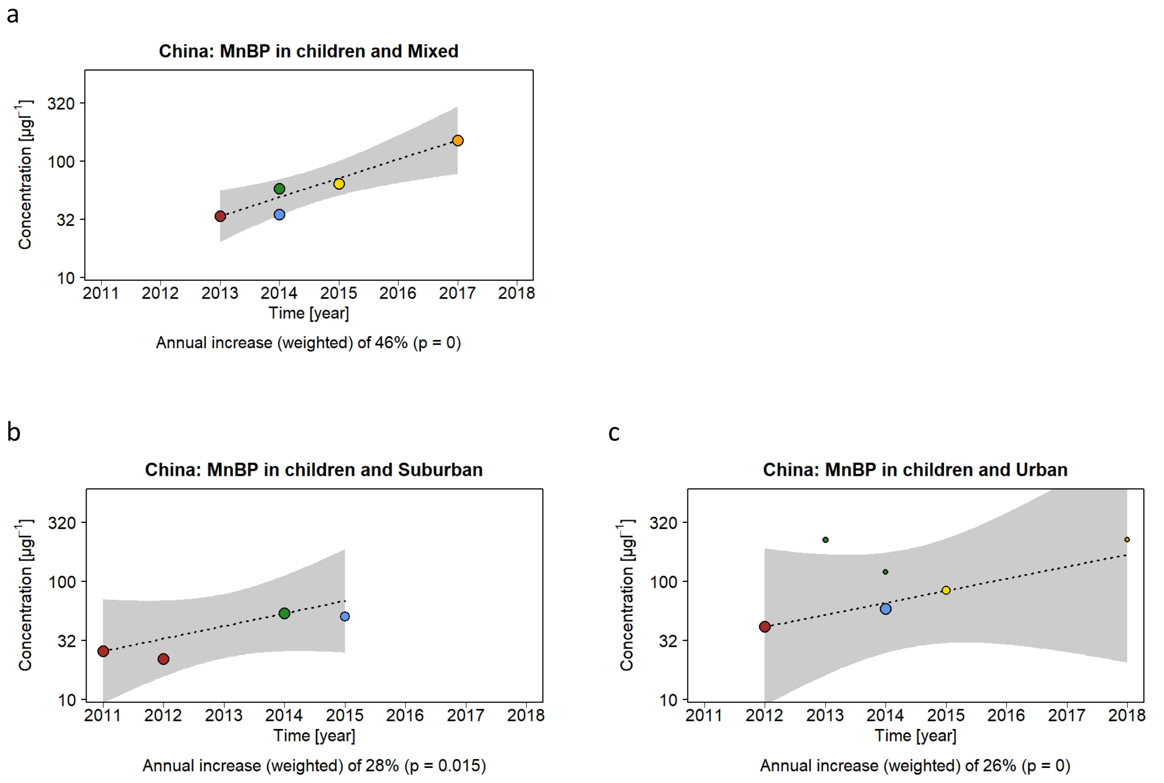

Figure S 10: significant time-trends for MnBP in Chinese children per area (a, mixed areas; b, suburban; c, urban)

Each symbol is a central value (i.e. median or geometric mean). The size of the symbols is related to the study-specific sample size (N) and its weight in the time-trend calculation (see Statistical methods, time-trend analysis). Within one panel, one colour identifies one Study ID number. However, in Figures S 8 to S 13, the colours are not comparable between panels and not comparable with the colours of other Figures in the manuscript. Grey area: 90% confidence interval. The p-value was < 0.001 for the trends shown in panels a and c

a

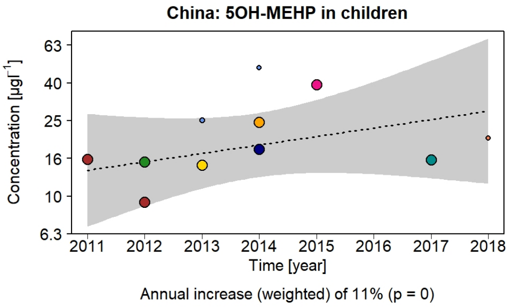

b

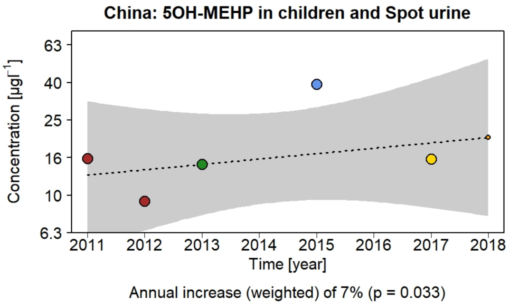

c

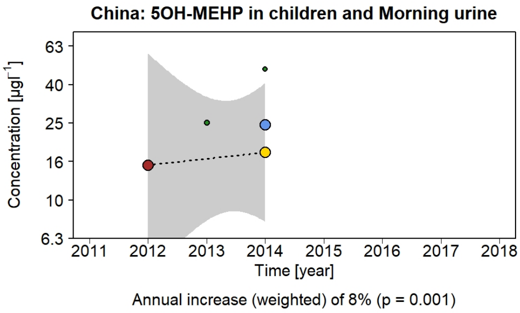

Figure S 11: time-trends for 5OH-MEHP in Chinese children: a) including all data; b) for data measured in spot urine; c) for data measured in morning urine

Each symbol is a central value (i.e. median or geometric mean). The size of the symbols is related to the study-specific sample size (N) and its weight in the time-trend calculation (see Statistical methods, time-trend analysis). Within one panel, one colour identifies one Study ID number. However, in Figures S 8 to S 13, the colours are not comparable between panels and not comparable with the colours of other Figures in the manuscript. Grey area: 90% confidence interval. The p-value was < 0.001 for the trend shown in panel a

a

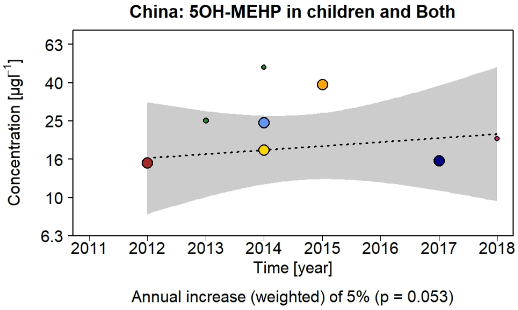

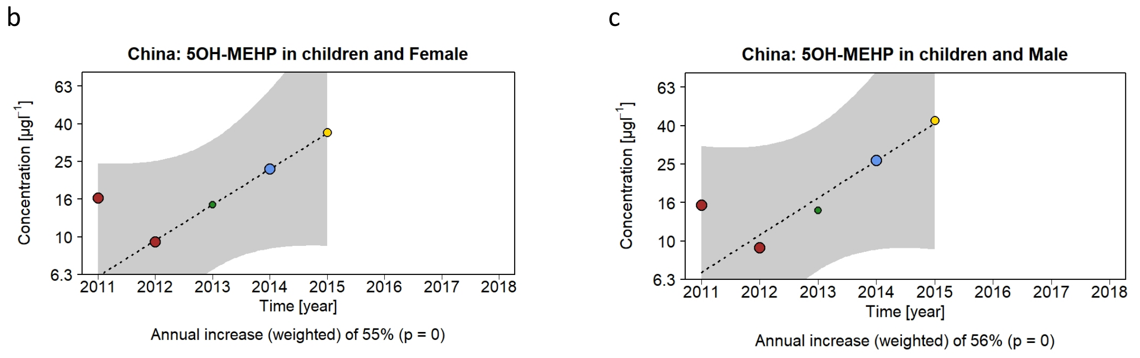

Figure S 12: significant time-trends for 5OH-MEHP in Chinese children per gender (a, both genders; b, girls; c, boys)

Each symbol is a central value (i.e. median or geometric mean). The size of the symbols is related to the study-specific sample size (N) and its weight in the time-trend calculation (see Statistical methods, time-trend analysis). Within one panel, one colour identifies one Study ID number. However, in Figures S 8 to S 13, the colours are not comparable between panels and not comparable with the colours of other Figures in the manuscript. Grey area: 90% confidence interval. The p-value was < 0.001 for the trends shown in panels b and c

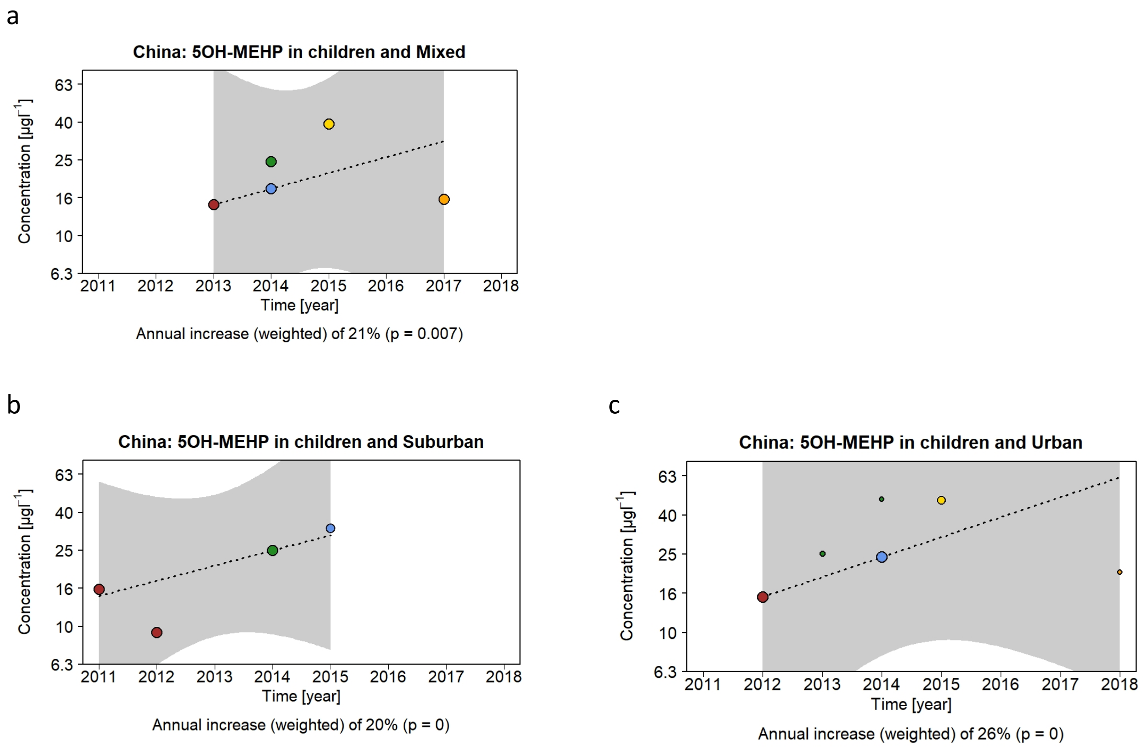

Figure S 13: significant time-trends for 5OH-MEHP in Chinese children per area (a, mixed areas; b, suburban; c, urban)

Each symbol is a central value (i.e. median or geometric mean). The size of the symbols is related to the study-specific sample size (N) and its weight in the time-trend calculation (see Statistical methods, time-trend analysis). Within one panel, one colour identifies one Study ID number. However, in Figures S 8 to S 13, the colours are not comparable between panels and not comparable with the colours of other Figures in the manuscript. Grey area: 90% confidence interval. The p-value was < 0.001 for the trends shown in panels b and c

a)

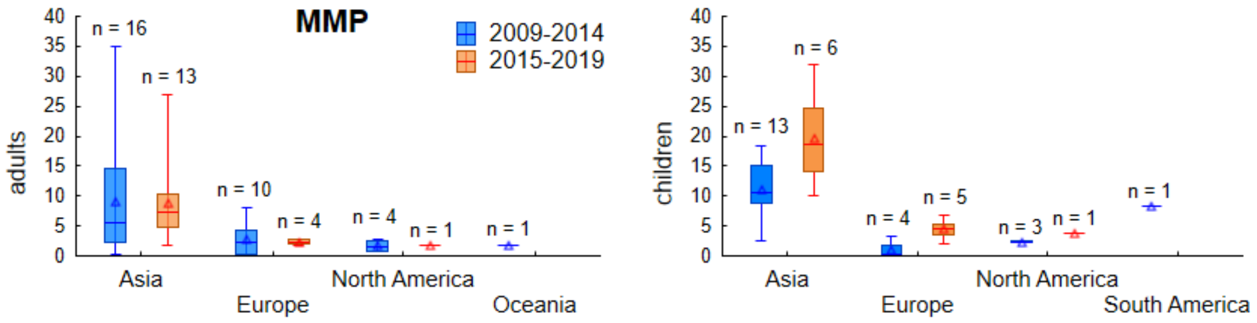
MMP
2009-2014
2015-2019
adults
children
0 5 10 15 20 25 30 35 40
n = 16
n = 13
n = 10
n = 4
n = 4
n = 1
n = 1
n = 6
n = 13
n = 4
n = 5
n = 3
n = 1
n = 1
Asia
Europe
North America
Oceania
South America

b)

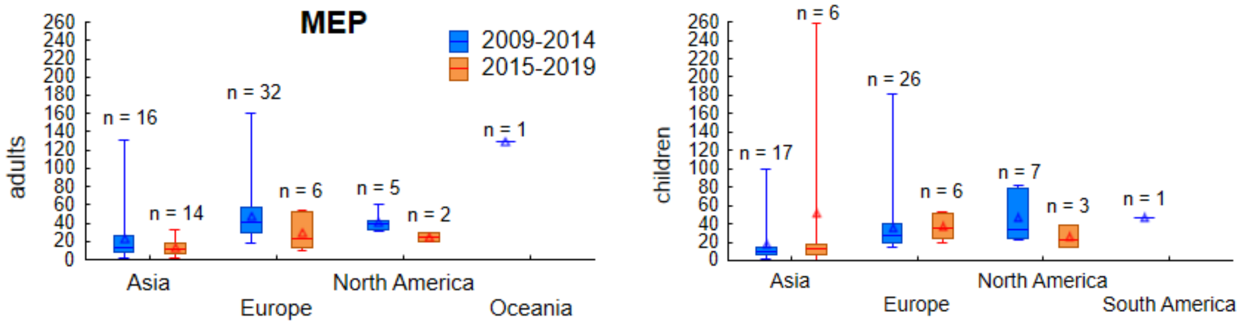
MEP
2009-2014
2015-2019
adults
children
0 20 40 60 80 100 120 140 160 180 200 220 240 260
n = 16
n = 14
n = 32
n = 6
n = 5
n = 2
n = 1
n = 6
n = 17
n = 26
n = 6
n = 7
n = 3
n = 1
Asia
Europe
North America
Oceania
South America

c)

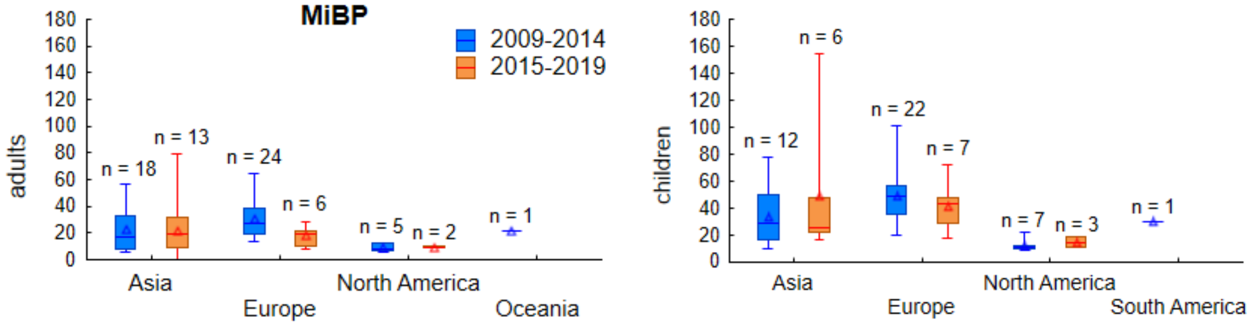
MiBP
2009-2014
2015-2019
adults
children
0 20 40 60 80 100 120 140 160 180
n = 18
n = 13
n = 24
n = 6
n = 5
n = 2
n = 1
n = 6
n = 12
n = 22
n = 7
n = 7
n = 3
n = 1
Asia
Europe
North America
Oceania
South America

d)

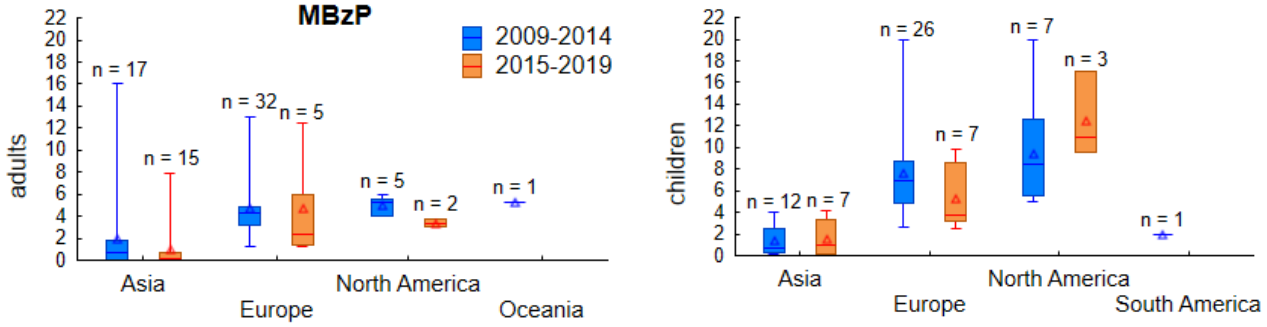
MBzP
2009-2014
2015-2019
adults
children
0 2 4 6 8 10 12 14 16 18 20 22
n = 17
n = 15
n = 32
n = 5
n = 5
n = 2
n = 1
n = 12
n = 7
n = 26
n = 7
n = 7
n = 3
n = 1
Asia
Europe
North America
Oceania
South America

e)

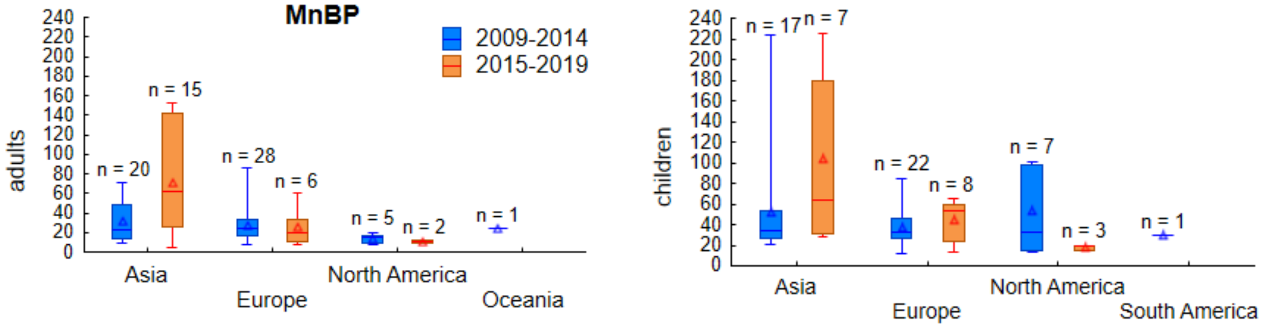

Figure S 14: boxplots of urinary concentrations (µg/L) of low-weight phthalates per continent and period (2009–2014 vs 2015–2019) in adults and children, for: MMP (a), MEP (b), MiBP (c), MBzP (d), and MnBP (e)

Triangular means average, line is median, box is 25-75th percentile range, whiskers are min.-max.

n is the number of central values per group. In the "PhthaLit" database, Egypt was classified as an Asian country.

a)

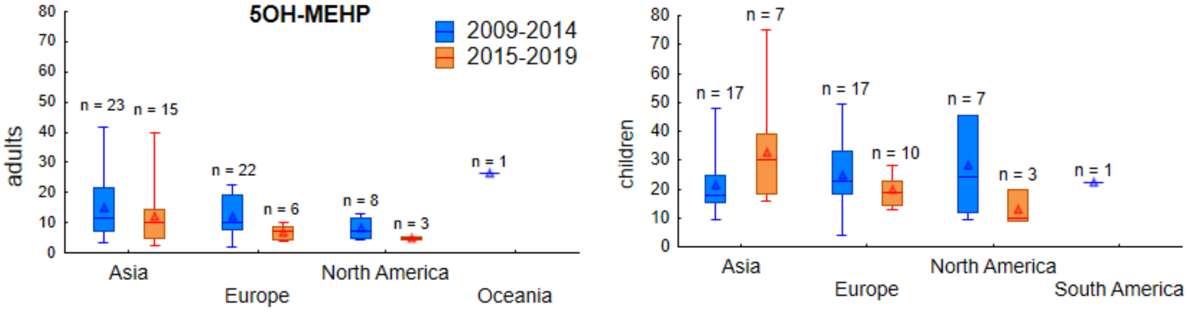

b)

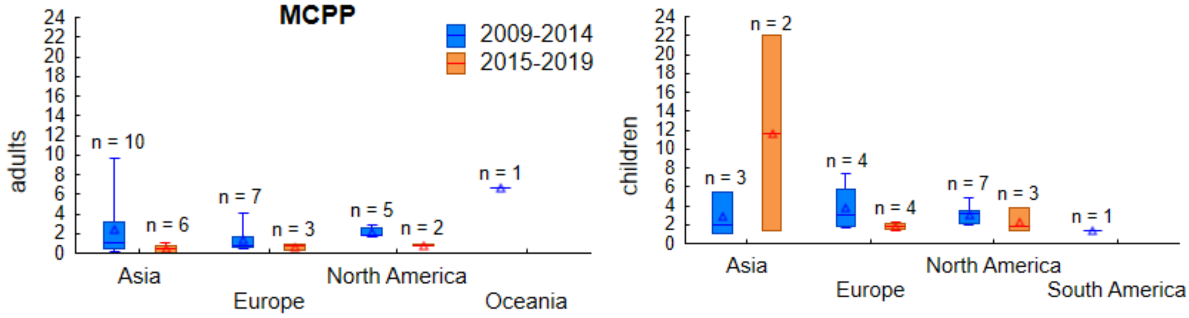

Figure S 15: boxplots of urinary concentrations (µg/L) per continent and period (2009–2014 vs 2015–2019) in adults and children, for: 5OH-MEHP (a), and MCPP (b)

Triangular means average, line is median, box is 25-75th percentile range, whiskers are min.-max. In the "PhthaLit" database, Egypt was classified as an Asian country.

S3. Additional information

S3.1. Urinary concentration units

For studies that reported only creatinine-corrected data, we aimed to back-calculate unadjusted values, by using Equation S 1:

Equation S 1 $$C_{\mathrm{Met(adj.Cr)}}\left(\frac{\mu\mathrm{g}}{\mathrm{g\ creatinine}}\right)\times C_{\mathrm{Creat}}\left(\frac{\mathrm{g\ creatinine}}{\mathrm{L}}\right)=C_{\mathrm{Met(unadj)}}\left(\frac{\mu\mathrm{g}}{\mathrm{L}}\right)$$

Where $C_{\mathrm{Met(adj.Cr)}}$ is the central value (median, geometric mean, GM) of creatinine-corrected concentration of metabolite in urine (µg/g creatinine), C_{Creat} is the central value (median, GM) of creatinine concentration in urine in the same samples (g creatinine/L), and $C_{\mathrm{Met(unadj)}}$ is the calculated unadjusted concentration of metabolite in urine (µg/L).

Among all studies in which phthalate concentrations were reported exclusively corrected by creatinine, one study in Taiwanese children by Su et al. (17) reported the geometric mean of creatinine concentrations measured in the same samples. Therefore, we back-calculated unadjusted phthalate concentrations for this study by using Equation S 1. Conversely, in other studies we were not able to find the median or GM value of measured creatinine concentrations. For the last studies, no unadjusted phthalate concentrations could be calculated and therefore, the reported data (in µg/g creatinine) were excluded from the data analysis.

S3.2. Correction of censored data

To deal with data below the limit of detection (LOD, left censoring) or below the limit of quantification (LOQ, interval censoring), different methods exist (18). For example, Helsel, 1990 (19) recommended maximum likelihood estimation (MLE) methods to estimate percentiles, and robust methods to estimate mean and standard deviation values. The selection of methods also depends on the percentage of censored data (18, 20).

In the publications from which censored data were compiled, substitution was the most frequent approach used to deal with censored data. Between substitution methods, replacing the data <LOD by either LOD/2 or by LOD/√2 made little difference in the estimates of geometric means by CDC (2). In our database, the central values reported to be <LOD or <LOQ were replaced by LOD/2 and LOQ/2, respectively. Few exceptions were made, for publications which reported non-detected central values (i.e. <LOD) and showed LOQ values rather than LOD values (21-25). In these specific cases, we replaced the central values <LOD by LOQ/2. Moreover in Lee I. et al. (23), the LOQ values for individual metabolites were not shown but they were reported to oscillate between 0.01 and 0.05 ng/mL. Therefore, to correct censored data in that publication, we considered an average LOQ value of 0.03 ng/mL.

S3.3. Impact of the gender, area, and sample type on the time-trends

In the literature, some HBM studies have found an impact of variables such as the gender, the sample type, or the area on phthalate concentrations in urine. Concerning the gender, we found no consensus in the literature about the possible impact of this variable on unadjusted phthalate concentrations in urine (µg/L). For example, some HBM studies have reported gender differences (34, 75), while others

have generally found no significant gender differences on the unadjusted phthalate concentrations (12, 13). Concerning the sample type, a relatively high within-day variability in phthalate concentrations in urine has been reported, with high concentrations in morning urine as compared to spot urine collected in the afternoon (76-78). Finally, some HBM studies have found differences between urban, suburban and/or rural areas in Asian countries (37, 79, 80). Between these variables, information on the gender and the sample type (spot urine, morning urine, 24-h urine) is generally shown in the HBM studies. Conversely, the area (urban, suburban, rural, mixed) is generally not specified. Therefore, for the analysis of the impact of the area on the time-trends, we focused specifically on China, where a high number of data sources was included in the time-trend analyses and a higher variability was expected. In summary, we analysed the impact of the gender and sample type on the time-trends in all countries, and the impact of the area on the time-trends in China. Specifically, all time-trend analyses were conducted separately per gender, area, and sample type, and compared to the original trends using Fisher z-scoring (81). None of these variables had a significant impact in any trend. The p-value of the trend difference test (81) was in all cases higher than 0.10.

For example, the global time-trends for MnBP in Chinese children (Figures 4b and S 8a) can be compared to the significant time-trends for MnBP in Chinese children calculated separately per sample type (Figure S 8b), gender (Figure S 9), and area (Figure S 10). Similarly, the global trend for 5OH-MEHP in Chinese children (Figure S 11 a) can be compared to the trends calculated separately per sample type (Figure S 11 b, c), gender (Figure S 12), and area (Figure S 13).

S3.4. Geographic comparisons

Geographic comparisons in the unadjusted urinary concentrations per substance, continent, and age group, were conducted. For these comparisons, we classified the data into two periods, specifically 2009–2014 and 2015–2019. Per parent substance, we selected either one monoester (for low-weight phthalates) or one secondary metabolite (for other phthalates and plasticizers). The selection of metabolites was also based on the number of data and the level of censoring (<=33%) per period and substance.

Different from the approach used for time-trend analysis, for geographic comparisons we included as variables: a) the continent (including Europe) instead of the country, and b) two sampling periods instead of study-specific sampling years. Also different from the time-trend analysis, for geographic comparisons we did not calculate the median value per metabolite, continent, age group, Study ID, and year. Therefore, to avoid duplicates in geographic comparisons, we excluded data reported for different subgroups (gender, area) of the same population (same Study ID and year).

Within one continent, the number of data differed between countries. For example, a high amount of data was available in China and Germany both for adults and children (Table S 3 and Table S 4) and, by consequence, data from these countries had a high weight within their continents. Interestingly, for some countries, a high amount of data existed principally either in adults (Denmark and Korea) or in children (e.g. Taiwan). Thus, if geographic differences were consistent between age groups, we considered these results to be important. Given the higher amount of data in Asia (As), Europe (Eu), and North America (NA), than in other continents, we compared the averages of central values in these three continents.

In brief, the averages of the compiled central values differed consistently between continents for both age groups and periods for: MMP (As > Eu and NA), MEP (Eu and NA > As), MiBP (Eu >= As > NA), and MBzP (NA > Eu > As) (Figure S 14, a-d). In Asia, for MnBP in adults and children, MMP and 5OH-MEHP

in children, the average concentrations increased from the first to the second period and were higher in this continent than in others for the second period (Figures S 14 a, e; S 15a). For other metabolites, either no consistent differences between continents for both age groups and periods were found (e.g. MCPP; Figure S 15b), or the number of data did not allow geographic comparisons (cx-MINP, OH-MINCH, 5OH-MEHTP; not shown).

References

1. Wang HX, Zhou Y, Tang CX, He YH, Wu JG, Chen Y, et al. Urinary Phthalate Metabolites Are Associated with Body Mass Index and Waist Circumference in Chinese School Children. Plos One. 2013;8(2).
2. CDC. Fourth National Report on Human Exposure to Environmental Chemicals, Updated Tables, January 2019, Volume One. U.S. Department of Health and Human Services. Centers for Disease Control and Prevention (CDC). 2019.
3. Frederiksen H, Nielsen O, Koch HM, Skakkebaek NE, Juul A, Jorgensen N, et al. Changes in urinary excretion of phthalates, phthalate substitutes, bisphenols and other polychlorinated and phenolic substances in young Danish men; 2009-2017. International Journal of Hygiene and Environmental Health. 2020;223(1):93-105.
4. Health_Canada. Fifth report on human biomonitoring of environmental chemicals in Canada. Results of the Canadian Health Measures Survey Cycle 5 (2016–2017), November 2019. 2019. p. 429 pp.
5. Koch HM, Ruther M, Schutze A, Conrad A, Palmke C, Apel P, et al. Phthalate metabolites in 24-h urine samples of the German Environmental Specimen Bank (ESB) from 1988 to 2015 and a comparison with US NHANES data from 1999 to 2012. International Journal of Hygiene and Environmental Health. 2017;220(2):130-41.
6. Cutanda F, Koch HM, Esteban M, Sanchez J, Angerer J, Castano A. Urinary levels of eight phthalate metabolites and bisphenol A in mother-child pairs from two Spanish locations. International Journal of Hygiene and Environmental Health. 2015;218(1):47-57.
7. Mullerova D, Bouchalova V, Matejkova D, Kovarova K, Svacina S, Vrbik K, et al. Phthalates exposure indicators determined by urinary phthalate metabolites in healthy non-obese Czech adults: FANTOM study. Food Additives and Contaminants Part A-Chemistry Analysis Control Exposure & Risk Assessment. 2016;33(12):1817-25.
8. Lee KM, Kho Y, Kim PG, Park SH, Lee JH. Urinary levels of phthalate metabolites and associations with demographic characteristics in Korean adults. Environmental Science and Pollution Research. 2017;24(17):14669-81.
9. Rocha BA, Asimakopoulos AG, Barbosa F, Kannan K. Urinary concentrations of 25 phthalate metabolites in Brazilian children and their association with oxidative DNA damage. Science of the Total Environment. 2017;586:152-62.
10. Schutze A, Gries W, Kolossa-Gehring M, Apel P, Schroter-Kermani C, Fiddicke U, et al. Bis-(2-propylheptyl)phthalate (DPHP) metabolites emerging in 24 h urine samples from the German Environmental Specimen Bank (1999-2012). International Journal of Hygiene and Environmental Health. 2015;218(6):559-63.
11. Gyllenhammar I, Glynn A, Jonsson BAG, Lindh CH, Darnerud PO, Svensson K, et al. Diverging temporal trends of human exposure to bisphenols and plastizisers, such as phthalates, caused by substitution of legacy EDCs? Environmental Research. 2017;153:48-54.
12. Schwedler G, Rucic E, Lange R, Conrad A, Koch HM, Palmke C, et al. Phthalate metabolites in urine of children and adolescents in Germany. Human biomonitoring results of the German

Environmental Survey GerES V, 2014-2017. International Journal of Hygiene and Environmental Health. 2020;225:11.
13. Schwedler, Gerda, Conrad A, Rucic E, Koch HM, Leng G, et al. Hexamoll® DINCH and DPHP metabolites in urine of children and adolescents in Germany. Human biomonitoring results of the German Environmental Survey GerES V, 2014–2017. International Journal of Hygiene and Environmental Health. 2020;229.
14. Kasper-Sonnenberg M, Koch HM, Apel P, Ruther M, Palmke C, Bruning T, et al. Time trend of exposure to the phthalate plasticizer substitute DINCH in Germany from 1999 to 2017: Biomonitoring data on young adults from the Environmental Specimen Bank (ESB). International Journal of Hygiene and Environmental Health. 2019;222(8):1084-92.
15. Silva MJ, Jia T, Samandar E, Preau JL, Calafat AM. Environmental exposure to the plasticizer 1,2-cyclohexane dicarboxylic acid, diisononyl ester (DINCH) in US adults (2000-2012). Environmental Research. 2013;126:159-63.
16. Langer S, Beko G, Weschler CJ, Brive LM, Toftum J, Callesen M, et al. Phthalate metabolites in urine samples from Danish children and correlations with phthalates in dust samples from their homes and daycare centers. International Journal of Hygiene and Environmental Health. 2014;217(1):78-87.
17. Su PH, Chen JY, Lin CY, Chen HY, Liao PC, Ying TH, et al. Sex Steroid Hormone Levels and Reproductive Development of Eight-Year-Old Children following In Utero and Environmental Exposure to Phthalates. Plos One. 2014;9(9).
18. Banta-Green CJ, Brewer AJ, Ort C, Helsel DR, Williams JR, Field JA. Using wastewater-based epidemiology to estimate drug consumption-Statistical analyses and data presentation. Science of the Total Environment. 2016;568:856-63.
19. Helsel DR. LESS THAN OBVIOUS - STATISTICAL TREATMENT OF DATA BELOW THE DETECTION LIMIT. Environmental Science & Technology. 1990;24(12):1766-74.
20. Asimakopoulos AG, Xue JC, De Carvalho BP, Iyer A, Abualnaja KO, Yaghmoor SS, et al. Urinary biomarkers of exposure to 57 xenobiotics and its association with oxidative stress in a population in Jeddah, Saudi Arabia. Environmental Research. 2016;150:573-81.
21. Guo Y, Alomirah H, Cho HS, Minh TB, Mohd MA, Nakata H, et al. Occurrence of Phthalate Metabolites in Human Urine from Several Asian Countries. Environmental Science & Technology. 2011;45(7):3138-44.
22. Guo Y, Wu Q, Kannan K. Phthalate metabolites in urine from China, and implications for human exposures. Environment International. 2011;37(5):893-8.
23. Lee I, Kim S, Park S, Mok S, Jeong Y, Moon HB, et al. Association of urinary phthalate metabolites and phenolics with adipokines and insulin resistance related markers among women of reproductive age. Science of the Total Environment. 2019;688:1319-26.
24. Yao Y, Chen DY, Wu Y, Zhou L, Cheng JQ, Ma YY, et al. Urinary phthalate metabolites in primary school starters in Pearl River Delta, China: Occurrences, risks and possible sources. Environmental Research. 2019;179.
25. Lee G, Kim S, Bastiaensen M, Malarvannan G, Poma G, Casero NC, et al. Exposure to organophosphate esters, phthalates, and alternative plasticizers in association with uterine fibroids. Environmental Research. 2020;189.
26. Choi W, Kim S, Baek YW, Choi K, Lee K, Do Yu S. Exposure to environmental chemicals among Korean adults-updates from the second Korean National Environmental Health Survey (2012-2014). International Journal of Hygiene and Environmental Health. 2017;220(2):29-35.
27. Park C, Hwang M, Baek Y, Jung S, Lee Y, Paek D, et al. Urinary phthalate metabolite and bisphenol A levels in the Korean adult population in association with sociodemographic and behavioral characteristics: Korean National Environmental Health Survey (KoNEHS) 2012-2014. International Journal of Hygiene and Environmental Health. 2019;222(5):903-10.
28. Park C, Hwang M, Kim H, Ryu S, Lee K, Choi K, et al. Early snapshot on exposure to environmental chemicals among Korean adults-results of the first Korean National Environmental

Health Survey (2009-2011). International Journal of Hygiene and Environmental Health. 2016;219(4-5):398-404.
29. Lim S. The associations between personal care products use and urinary concentrations of phthalates, parabens, and triclosan in various age groups: The Korean National Environmental Health Survey Cycle 3 2015-2017. Science of the Total Environment. 2020;742.
30. Kang H, Kim S, Lee G, Lee I, Lee JP, Lee J, et al. Urinary metabolites of dibutyl phthalate and benzophenone-3 are potential chemical risk factors of chronic kidney function markers among healthy women. Environment International. 2019;124:354-60.
31. Chen JS, Zhou XF, Zhang H, Liu YM, Cao C, Dong RH, et al. Association between urinary concentration of phthalate metabolites and impaired renal function in Shanghai adults. Environmental Pollution. 2019;245:149-62.
32. Dong RH, Zheng JH, Zhang MR, Chen JS, Zhang H, Gao X, et al. The concentrations and cumulative risk assessment of phthalates in general population from Shanghai: The comparison between groups with different ages. Science of the Total Environment. 2018;637:871-80.
33. Liao CX, Liu W, Zhang JL, Shi WM, Wang XY, Cai J, et al. Urine Metabolites of Phthalate Esters in 434 Shanghai Children and Their Associations with Ventilation Habits. 10th International Symposium on Heating, Ventilation and Air Conditioning, Ishvac2017. 2017;205:1146-51.
34. Liao CX, Liu W, Zhang JL, Shi WM, Wang XY, Cai J, et al. Associations of urinary phthalate metabolites with residential characteristics, lifestyles, and dietary habits among young children in Shanghai, China. Science of the Total Environment. 2018;616:1288-97.
35. Yao Y, Chen DY, Yin JW, Zhou L, Cheng JQ, Lu SY, et al. Phthalate exposure linked to high blood pressure in Chinese children. Environment International. 2020;143:8.
36. Huang HB, Siao CY, Lo YTC, Shih SF, Lu CH, Huang PC. Mediation effects of thyroid function in the associations between phthalate exposure and glucose metabolism in adults. Environmental Pollution. 2021;278:10.
37. Liao KW, Chang WH, Chou WC, Huang HB, Waits A, Chen PC, et al. Human biomonitoring reference values and characteristics of Phthalate exposure in the general population of Taiwan: Taiwan Environmental Survey for Toxicants 2013-2016. International Journal of Hygiene and Environmental Health. 2021;235.
38. Goodrich JM, Dolinoy DC, Sanchez BN, Zhang ZZ, Meeker JD, Mercado-Garcia A, et al. Adolescent epigenetic profiles and environmental exposures from early life through peri-adolescence. Environmental Epigenetics. 2016;2(3).
39. Lewis RC, Meeker JD, Peterson KE, Lee JM, Pace GG, Cantoral A, et al. Predictors of urinary bisphenol A and phthalate metabolite concentrations in Mexican children. Chemosphere. 2013;93(10):2390-8.
40. Feng YL, Liao XJ, Chen D, Takser L, Cakmak S, Chan P, et al. Correlations of phthalate metabolites in urine samples from fertile and infertile men: Free-form concentration vs. conjugated-form concentration. Environmental Pollution. 2020;263:9.
41. Albert O, Huang JY, Aleksa K, Hales BF, Goodyer CG, Robaire B, et al. Exposure to polybrominated diphenyl ethers and phthalates in healthy men living in the greater Montreal area: A study of hormonal balance and semen quality. Environment International. 2018;116:165-75.
42. Tang SY, He C, Thai P, Vijayasarathy S, Mackie R, Toms LML, et al. Concentrations of phthalate metabolites in Australian urine samples and their contribution to the per capita loads in wastewater. Environment International. 2020;137:8.
43. Gómez-Ramos MJ, Heffernan AL, Toms LML, Calafat AM, Ye X, Hobson P, et al. Concentrations of phthalates and DINCH metabolites in pooled urine from Queensland, Australia. Environment International. 2016;88:179-86.
44. Schoeters G, Govarts E, Bruckers L, Den Hond E, Nelen V, De Henauw S, et al. Three cycles of human biomonitoring in Flanders - Time trends observed in the Flemish Environment and Health Study. International Journal of Hygiene and Environmental Health. 2017;220(2):36-45.

45. Koppen G, Franken C, Den Hond E, Plusquin M, Reimann B, Leermakers M, et al. Pooled analysis of genotoxicity markers in relation to exposure in the Flemish Environment and Health Studies (FLEHS) between 1999 and 2018. Environmental Research. 2020;190:13.
46. Schwedler G, Rucic E, Koch HM, Lessmann F, Bruning T, Conrad A, et al. Metabolites of the substitute plasticiser Di-(2-ethylhexyl) terephthalate (DEHTP) in urine of children and adolescents investigated in the German Environmental Survey GerES V, 2014-2017. International Journal of Hygiene and Environmental Health. 2020;230.
47. Schmidtkunz C, Gries W, Weber T, Leng G, Kolossa-Gehring M. Internal exposure of young German adults to di(2-propylheptyl) phthalate (DPHP): Trends in 24-h urine samples from the German Environmental Specimen Bank 1999-2017. International Journal of Hygiene and Environmental Health. 2019;222(3):419-24.
48. Lessmann F, Kolossa-Gehring M, Apel P, Ruther M, Palmke C, Harth V, et al. German Environmental Specimen Bank: 24-hour urine samples from 1999 to 2017 reveal rapid increase in exposure to the para-phthalate plasticizer di(2-ethylhexyl) terephthalate (DEHTP). Environment International. 2019;132.
49. Schutze A, Kolossa-Gehring M, Apel P, Bruning T, Koch HM. Entering markets and bodies: Increasing levels of the novel plasticizer Hexamoll (R) DINCH (R) in 24 h urine samples from the German Environmental Specimen Bank. International Journal of Hygiene and Environmental Health. 2014;217(2-3):421-6.
50. Fromme H, Lahrz T, Kraft M, Fembacher L, Dietrich S, Sievering S, et al. Phthalates in German daycare centers: Occurrence in air and dust and the excretion of their metabolites by children (LUPE 3). Environment International. 2013;61:64-72.
51. Fromme H, Lahrz T, Kraft M, Fembacher AL, Schutze A, Dietrich S, et al. Exposure to plasticizers in German daycare centers: the LUPE 3 study. Air Pollution Xxiii. 2015;198:369-76.
52. Fromme H, Lahrz T, Kraft M, Fembacher L, Mach C, Dietrich S, et al. Organophosphate flame retardants and plasticizers in the air and dust in German daycare centers and human biomonitoring in visiting children (LUPE 3). Environment International. 2014;71:158-63.
53. Den Hond E, Govarts E, Willems H, Smolders R, Casteleyn L, Kolossa-Gehring M, et al. First Steps toward Harmonized Human Biomonitoring in Europe: Demonstration Project to Perform Human Biomonitoring on a European Scale. Environmental Health Perspectives. 2015;123(3):255-63.
54. Koppen G, Govarts E, Vanermen G, Voorspoels S, Govindan M, Dewolf MC, et al. Mothers and children are related, even in exposure to chemicals present in common consumer products. Environmental Research. 2019;175:297-307.
55. Cerna M, Maly M, Rudnai P, Kozepesy S, Naray M, Halzlova K, et al. Case study: Possible differences in phthalates exposure among the Czech, Hungarian, and Slovak populations identified based on the DEMOCOPHES pilot study results. Environmental Research. 2015;141:118-24.
56. Schwedler G, Seiwert M, Fiddicke U, Issleb S, Holzer J, Nendza J, et al. Human biomonitoring pilot study DEMOCOPHES in Germany: Contribution to a harmonized European approach. International Journal of Hygiene and Environmental Health. 2017;220(4):686-96.
57. Cullen E, Evans D, Griffin C, Burke P, Mannion R, Burns D, et al. Urinary Phthalate Concentrations in Mothers and Their Children in Ireland: Results of the DEMOCOPHES Human Biomonitoring Study. International Journal of Environmental Research and Public Health. 2017;14(12).
58. Runkel AA, Snoj-Tratnik J, Mazej D, Horvat M. Urinary phthalate concentrations in the slovenian population: An attempt to exposure assessment of family units. Environmental Research. 2020;186:13.
59. Larsson K, Bjorklund KL, Palm B, Wennberg M, Kaj L, Lindh CH, et al. Exposure determinants of phthalates, parabens, bisphenol A and triclosan in Swedish mothers and their children. Environment International. 2014;73:323-33.
60. Exley K, Aerts D, Biot P, Casteleyn L, Kolossa-Gehring M, Schwedler G, et al. Pilot study testing a European human biomonitoring framework for biomarkers of chemical exposure in

children and their mothers: experiences in the UK. Environmental Science and Pollution Research. 2015;22(20):15821-34.
61. Myridakis A, Fthenou E, Balaska E, Vakinti M, Kogevinas M, Stephanou EG. Phthalate esters, parabens and bisphenol-A exposure among mothers and their children in Greece (Rhea cohort). Environment International. 2015;83:1-10.
62. Myridakis A, Chalkiadaki G, Fotou M, Kogevinas M, Chatzi L, Stephanou EG. Exposure of Preschool-Age Greek Children (RHEA Cohort) to Bisphenol A, Parabens, Phthalates, and Organophosphates. Environmental Science & Technology. 2016;50(2):932-41.
63. Haug LS, Sakhi AK, Cequier E, Casas M, Maitre L, Basagana X, et al. In-utero and childhood chemical exposome in six European mother-child cohorts. Environment International. 2018;121:751-63.
64. Tranfo G, Papaleo B, Caporossi L, Capanna S, De Rosa M, Pigini D, et al. Urinary metabolite concentrations of phthalate metabolites in Central Italy healthy volunteers determined by a validated HPLC/MS/MS analytical method. International Journal of Hygiene and Environmental Health. 2013;216(4):481-5.
65. Tranfo G, Caporossi L, Pigini D, Capanna S, Papaleo B, Paci E. Temporal Trends of Urinary Phthalate Concentrations in Two Populations: Effects of REACH Authorization after Five Years. International Journal of Environmental Research and Public Health. 2018;15(9).
66. Giovanoulis G, Alves A, Papadopoulou E, Cousins AP, Schutze A, Koch HM, et al. Evaluation of exposure to phthalate esters and DINCH in urine and nails from a Norwegian study population. Environmental Research. 2016;151:80-90.
67. Alves A, Giovanoulis G, Nilsson U, Erratico C, Lucattini L, Haug LS, et al. Case Study on Screening Emerging Pollutants in Urine and Nails. Environmental Science & Technology. 2017;51(7):4046-53.
68. Milosevic N, Milic N, Bosic DZ, Bajkin I, Percic I, Abenavoli L, et al. Potential influence of the phthalates on normal liver function and cardiometabolic risk in males. Environmental Monitoring and Assessment. 2018;190(1).
69. Milosevic N, Milanovic M, Sudji J, Zivanovic DB, Stojanoski S, Vukovic B, et al. Could phthalates exposure contribute to the development of metabolic syndrome and liver disease in humans? Environmental Science and Pollution Research. 2020;27(1):772-84.
70. Polanska K, Ligocka D, Sobala W, Hanke W. Phthalate exposure and child development: The Polish Mother and Child Cohort Study. Early Human Development. 2014;90(9):477-85.
71. Gari M, Koch HM, Palmke C, Jankowska A, Wesolowska E, Hanke W, et al. Determinants of phthalate exposure and risk assessment in children from Poland. Environment International. 2019;127:742-53.
72. Correia-Sa L, Schutze A, Norberto S, Calhau C, Domingues VF, Koch HM. Exposure of Portuguese children to the novel non-phthalate plasticizer di-(iso-nonyl)-cyclohexane-1,2-dicarboxylate (DINCH). Environment International. 2017;102:79-86.
73. Correia-Sa L, Kasper-Sonnenberg M, Palmke C, Schutze A, Norberto S, Calhau C, et al. Obesity or diet? Levels and determinants of phthalate body burden - A case study on Portuguese children. International Journal of Hygiene and Environmental Health. 2018;221(3):519-30.
74. Lessmann F, Correia-Sa L, Calhau C, Domingues VF, Weiss T, Bruning T, et al. Exposure to the plasticizer di(2-ethylhexyl) terephthalate (DEHTP) in Portuguese children -Urinary metabolite levels and estimated daily intakes. Environment International. 2017;104:25-32.
75. Ding S, Zhang ZM, Chen YJ, Qi W, Zhang YZ, Xu Q, et al. Urinary levels of phthalate metabolites and their association with lifestyle behaviors in Chinese adolescents and young adults. Ecotoxicology and Environmental Safety. 2019;183.
76. Sakhi AK, Sabaredzovic A, Cequier E, Thomsen C. Phthalate metabolites in Norwegian mothers and children: Levels, diurnal variation and use of personal care products. Science of the Total Environment. 2017;599:1984-92.

77. Fays F, Palazzi P, Hardy EM, Schaeffer C, Phillipat C, Zeimet E, et al. Is there an optimal sampling time and number of samples for assessing exposure to fast elimination endocrine disruptors with urinary biomarkers? Science of the Total Environment. 2020;747.
78. Bastiaensen M, Malarvannan G, Gys C, Bamai YA, Araki A, Covaci A. Between- and within-individual variability of urinary phthalate and alternative plasticizer metabolites in spot, morning void and 24-h pooled urine samples. Environmental Research. 2020;191:11.
79. Li XJ, Zhong Y, He WY, Huang SY, Li Q, Guo CS, et al. Co-exposure and health risks of parabens, bisphenols, triclosan, phthalate metabolites and hydroxyl polycyclic aromatic hydrocarbons based on simultaneous detection in urine samples from guangzhou, south China. Environmental Pollution. 2021;272.
80. Yu YX, Peng MM, Liu YL, Ma JJ, Wang N, Ma ST, et al. Co-exposure to polycyclic aromatic hydrocarbons and phthalates and their associations with oxidative stress damage in school children from South China. Journal of Hazardous Materials. 2021;401.
81. Fisher RA. Frequency Distribution of the Values of the Correlation Coefficient in Samples from an Indefinitely Large Population. Biometrika. 1915;10(4):507-21.
